# Mechanism of periocular acupuncture in alleviating dry eye neuropathic pain via regulation of the “periocular acupoint–trigeminal ganglion–ventral posteromedial thalamic nucleus” pathway

**DOI:** 10.3389/fmed.2026.1803621

**Published:** 2026-05-05

**Authors:** Xinchen Wu, Qingbo Wei, Wenwen Zhang, Dandan Zhu, Weiping Gao, Ning Ding

**Affiliations:** 1 Department of Ophthalmology, Affiliated Hospital of Nanjing University of Chinese Medicine, Nanjing, Jiangsu, China; 2 School of Acupuncture-Moxibustion and Tuina, Nanjing University of Chinese Medicine, Nanjing, Jiangsu, China; 3 Department of Ophthalmology, Nanjing Drum Tower Hospital, The Affiliated Hospital of Nanjing University Medical School, Nanjing, Jiangsu, China

**Keywords:** acupuncture, dry eye neuropathic pain, periocular acupoints, trigeminal nerve, ventral posteromedial thalamic nucleus

## Abstract

**Objective:**

Dry eye disease (DED) is a common ocular surface disorder characterized by tear film instability and trigeminal nerve-mediated corneal hyperesthesia. Periocular acupuncture has been demonstrated to relieve DED symptoms; however, the underlying mechanisms remain unclear. Thus, in this study, we aimed to investigate the therapeutic effect of periocular acupuncture on DED and its peripheral-central regulatory mechanisms.

**Materials and methods:**

A guinea pig model of dry eye neuropathic pain was established through subcutaneous injection of scopolamine hydrobromide combined with topical instillation of benzalkonium chloride solution. After 21 days, the model animals were treated with either periocular acupoint acupuncture (Acu) or non-acupoint acupuncture (NA). Dry eye-related parameters and ocular surface sensitivity indicators were measured. Histopathological changes in the ocular surface, lacrimal glands, and trigeminal ganglia (TG) were examined using hematoxylin–eosin staining, *in vivo* confocal microscopy, and transmission electron microscopy. Neural tracing was performed using Alexa Fluor 488 Cholera Toxin Subunit B (CTB-488) and Fluoro-Gold (FG) tracers, and patch-clamp recordings on *ex vivo* brain slices were utilized to assess the modulatory effects of periocular acupuncture on the excitability of ventral posteromedial thalamic nucleus (VPM) neurons.

**Results:**

Periocular acupuncture effectively improved dry eye symptoms and abnormal pain sensations. Additionally, it ameliorated lacrimal gland atrophy, reduced corneal epithelial compensatory hyperplasia and inflammatory cell infiltration, and improved the structural disorganization and pyknosis of the subbasal corneal nerve plexus and TG neurons. Neural tracing results revealed that the Acu group, particularly at TaiYang (EX-HN5) and JingMing (BL1) acupoints, specifically targeted the TG–VPM neural pathway, with significantly higher numbers of labeled neurons and stronger fluorescence intensity than the NA group (*P* < 0.01). Patch-clamp recordings demonstrated that periocular acupuncture suppressed the hyperexcitability of VPM neurons in the model group, as reflected by decreased action potential firing and increased rheobase.

**Conclusion:**

Our results support the existence of a specific “periocular acupoint–TG–VPM” neural pathway, identify the pathological feature of VPM neuron hyperexcitability in dry eye neuropathic pain, and suggest that periocular acupuncture alleviates dry eye-related pain partially through regulating neuronal excitability. These findings provide experimental evidence for the clinical application.

## 1 Introduction

DED is a prevalent ocular surface disorder with a global prevalence ranging from 5.4 to 44.2% (1). Recent studies have shown that 82% of patients with DED experience pain symptoms (2), mainly manifesting as ocular pain, burning sensation, and foreign body sensation, which severely impact their quality of life.

Previous studies have indicated that ocular pain signals are transmitted to the trigeminal nerve, and dry eye neuropathic pain induced by long-term DED is mediated by this neural pathway (3, 4). As the primary nerve responsible for ocular surface sensation, the ophthalmic branch (V1) of the trigeminal nerve has dense peripheral nerve fibers distributed in the cornea and conjunctiva, forming an extensive nerve plexus. Notably, the cornea is innervated by the most abundant nerve fibers among all ocular structures (5–7). When harmful stimuli (such as mechanical or chemical stimuli) or inflammatory mediators induced by conditions, including DED, act on the cornea, they activate pain receptors on the trigeminal nerve terminals (8). These receptors express various ion channels (e.g., TRPV1, ASIC, and Nav1.8) (9–13), which can convert stimulus energy into electrical signals. These electrical signals are transmitted as action potentials to the central nervous system via the TG, undergo initial sensory processing in the spinal trigeminal nucleus (Sp5), and subsequently project to the VPM (14, 15). As a key relay station in the trigeminal sensory pathway, the VPM receives sensory inputs from the face and eyes and transmits them to the cerebral cortex (16, 17). Studies have found that long-term peripheral inflammation can lead to central sensitization of thalamic neurons, and the hyperexcitability of VPM neurons ultimately causes hyperalgesia and allodynia (18).

Currently, commonly used clinical treatments, including artificial tears and anti-inflammatory drugs, can alleviate ocular surface symptoms but have limited efficacy in treating neuropathic pain (19). Acupuncture, a key component of traditional Chinese medicine, exerts positive effects in analgesia and regulation of neuronal plasticity. It can relieve neuropathic pain by modulating central neuronal excitability, achieving longer-lasting analgesic effects (20–23). Clinical studies have confirmed that acupuncture exhibits above-average efficacy in treating chronic pains, including cephalic and facial, neck-shoulder, and low back pains (24, 25). Furthermore, our previous studies have demonstrated the unique advantages of acupuncture in treating dry eye neuropathic pain: periocular acupuncture not only improves ocular surface damage and reduces ocular inflammation but also alleviates pain sensations by repairing sensory nerve pathway injuries (26–28). However, the central mechanism of periocular acupuncture in treating dry eye neuropathic pain remains unclear. Employing a multidisciplinary research strategy, this study integrated neural tracing technology and electrophysiological recording techniques to explore the therapeutic effect of periocular acupuncture on DED and its peripheral-central regulatory mechanisms.

## 2 Materials and methods

### 2.1 Experimental animals

Twelve-week-old healthy male albino guinea pigs, weighing 350 ± 50 g, were obtained from Dongfang Breeding Co., Ltd., Animal Production License No.: SCXK (Su) 2022-0004. The animals were housed in the Basic Pharmacology Laboratory of the Affiliated Hospital of Nanjing University of Chinese Medicine (Animal Use License No.: SYXK (Su) 2022-0069) under controlled temperature and humidity, with a 12-h light/dark cycle, and were provided ad libitum access to food and water. The Experimental Animal Ethics Committee of the Affiliated Hospital of Nanjing University of Chinese Medicine approved the experimental protocol (batch number: 2024DW-038-02).

### 2.2 Establishment of the dry eye neuropathic pain model

**Figure 1:**
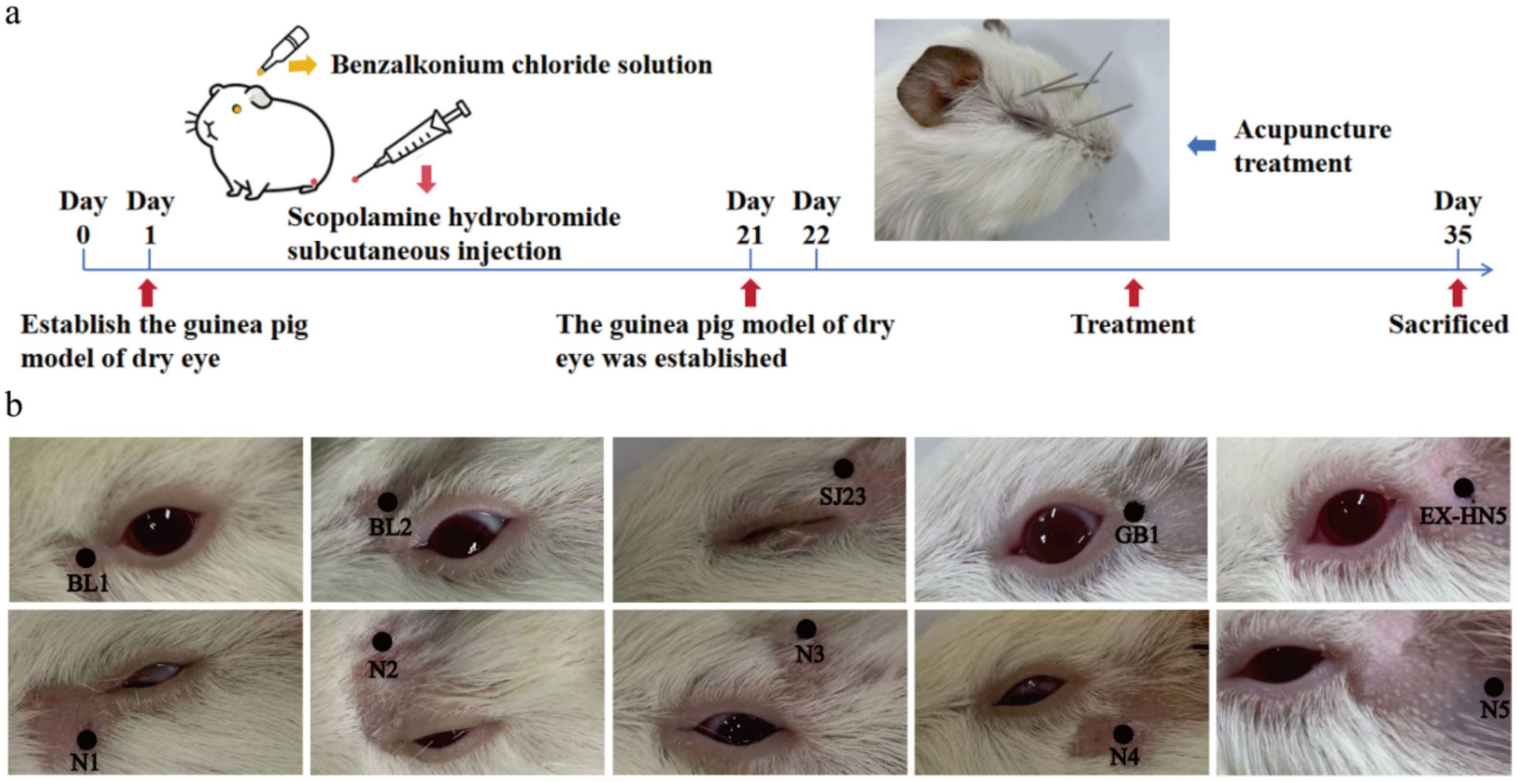
Establishment of the guinea pig dry eye model and acupuncture intervention protocol. **(a)** Flowchart of dry eye model establishment: The guinea pig model of dry eye neuropathic pain was established and maintained through subcutaneous injection of scopolamine hydrobromide combined with topical instillation of benzalkonium chloride for 35 days. Acupuncture treatment was administered to the Acu group from day 22 to day 35. **(b)** Periocular acupoints in the Acu group included: JingMing (BL1): depression on the medial orbital wall superior to the medial canthus; CuanZhu (BL2): frontal notch on the medial upper orbit; SiZhukong (SJ23): depression on the lateral upper orbit; TongZiliao (GB1): depression lateral to the lateral canthus; and TaiYang (EX-HN5): approximately 0.5 cm posterior to the lateral canthus. Non-acupoint areas in the NA group included: N1: 0.5 cm directly below JingMing (BL1); N2: 0.5 cm directly above CuanZhu (BL2); N3: 0.5 cm directly above SiZhukong (SJ23); N4: 0.5 cm directly below TongZiliao (GB1); and N5: 0.5 cm directly behind TaiYang (EX-HN5).

Guinea pigs received subcutaneous injections of scopolamine hydrobromide solution (Chengdu Pufeide Biotechnology Co., Ltd., China) at a dose of 0.2 mL per injection (2.5 mg/mL) in the right hind limb four times daily, combined with topical instillation of 0.2% benzalkonium chloride solution (Nanjing Wanqing Chemical Glass Instrument Co., Ltd., China) in both eyes three times daily for 35 consecutive days (Figure 1a) (29).

### 2.3 Intervention treatments

Control group (Con): Guinea pigs received subcutaneous injections of 0.2 mL of 0.9% sodium chloride solution (Otsuka Pharmaceutical Co., Ltd., China) in the right hind limb four times daily, combined with topical instillation of 0.9% sodium chloride solution in both eyes three times daily for 35 consecutive days.

Model group (Mod): Guinea pigs maintained the dry eye neuropathic pain model without any therapeutic intervention.

Periocular acupoint acupuncture group (Acu): Starting from day 22 after model establishment, guinea pigs in the dry eye neuropathic pain model received acupuncture treatment. During acupuncture, the animals were restrained but remained awake. The bilateral acupoints included the following: JingMing (BL1, needled obliquely inward and downward 3 mm subcutaneously), CuanZhu (BL2, needled horizontally downward 3 mm), SiZhukong (SJ23, needled horizontally from anterior to posterior 3 mm), TongZiliao (GB1, needled horizontally outward 3 mm), and TaiYang (EX-HN5, needled vertically 3 mm). The needles were retained for 15 min, once a day, for 14 consecutive days.

Non-acupoint acupuncture group (NA): Starting from day 22 after model establishment, guinea pigs in the dry eye neuropathic pain model received acupuncture at non-acupoint areas. During acupuncture, the animals were restrained but remained awake. The bilateral non-acupoint areas included: 0.5 cm directly below BL1 (needled obliquely inward and downward 3 mm subcutaneously), 0.5 cm directly above BL2 (needled horizontally downward 3 mm), 0.5 cm directly above SJ23 (needled horizontally from anterior to posterior 3 mm), 0.5 cm directly below GB1 (needled horizontally outward 3 mm), and 0.5 cm directly behind EX-HN5 (needled vertically 3 mm). The needles were retained for 15 min, once a day, for 14 consecutive days.

Disposable sterile acupuncture needles (model: 0.18 × 13 mm, Suzhou Medical Instrument Factory, China) were used. Guinea pigs in the Con and Mod groups were only restrained for the same duration without any intervention (Figures 1a,b).

### 2.4 Assessment of dry eye neuropathic pain-related parameters

Dry eye neuropathic pain-related parameters were measured in all groups on days 0 (before modeling), 21 (before intervention), and 35 (after intervention). Additionally, *in vivo* confocal microscopy (IVCM) examination was performed on day 35 after intervention. All measurements were performed in awake and quiet guinea pigs without the use of sedatives or anesthetics, and were conducted strictly in order from non-contact to contact and from non-invasive to invasive, with an interval of at least 5 min between tests to ensure ocular surface recovery and minimize interference. After completing all examinations, the guinea pigs were anesthetized with 3% isoflurane (Shenzhen RWD Life Science Co., Ltd., China) through inhalation and then sacrificed by neck removal. According to the double-blind principle, one researcher without knowledge of the group allocation collected the data, and another researcher analyzed the data.

#### 2.4.1 Blinking frequency

Video recording was used to count the number of blinks in the guinea pig within 5 min in a quiet state.

#### 2.4.2 Palpebral fissure height

The palpebral fissure height was measured as the vertical distance from the midpoint of the upper eyelid margin to the midpoint of the lower eyelid margin by digital caliper when the guinea pig was in a quiet and naturally open-eyed state, with an accuracy of 0.5 mm.

#### 2.4.3 Tear film break-up time

A moistened corneal staining filter strip (Tianjin Jingming New Technology Development Co., Ltd., China) was gently touched to the inferior conjunctival fornix. The time from the last blink to the appearance of the first dry corneal spot was recorded under a slit-lamp microscope, with each measurement repeated three times.

#### 2.4.4 Corneal fluorescein staining score

Fluorescein sodium ophthalmic test strips (Tianjin Jingming New Technology Development Co., Ltd., China) were moistened with 0.9% sodium chloride solution, and the stained tip was gently applied to the inner surface of the lower eyelid of one eye. After the guinea pig blinked several times, the corneal epithelium was observed under cobalt blue light illumination using a slit-lamp microscope. Epithelial lesions appeared as punctate or flaky green fluorescence, while intact epithelium showed blue fluorescence. Each corneal quadrant was scored from 0 to 3: 0, 1, 2, and 3 points for no staining, <5 punctate stains, >5 punctate stains, and flaky staining, respectively. The total score was the sum of scores from the four quadrants.

#### 2.4.5 Phenol red thread test

One end of a phenol red thread (Tianjin Jingming New Technology Development Co., Ltd., China) was folded and placed in the lateral one-third of the inferior conjunctival sac. After 20 s, the thread was removed, and the wet length was measured from the folded end.

#### 2.4.6 Corneal mechanical sensitivity

Threshold: The nylon filament of a corneal sensitivity tester (Luneau Technology, France) was adjusted to 60 mm. The tester was held by hand, and the tip of the nylon filament was gently applied vertically to one cornea to observe the blink reflex. The test was repeated three times at the same length; if blinking occurred ≥2 times, the length of the nylon filament was recorded as the corneal mechanical sensitivity threshold for that eye. If no blinking occurred, the length of the nylon filament was shortened by 5 mm each time, and the above steps were repeated until blinking occurred ≥2 times. The corneal mechanical sensitivity thresholds were measured at the central, superior, inferior, nasal, and temporal regions of the cornea.

#### 2.4.7 IVCM

Guinea pigs received daily adaptive handling and restraint training for 1 week prior to examination to reduce stress responses and motion artifacts during imaging. Examination was immediately terminated if the animals exhibited obvious struggling or restlessness. Topical anesthesia was administered with oxybuprocaine hydrochloride eye drops (Santen Pharmaceutical Co., Ltd., China). The guinea pig body was gently restrained in a cloth bag and head was fixed in front of a confocal microscope (HRT III RCM, Heidelberg Engineering, Germany). Vidisic transparent gel (Bausch & Lomb, United States) was applied to the surface of a 40× water-immersion objective lens. The lens was advanced slowly, and the recording button was pressed when corneal cells were visible in the center of the monitor screen to capture images of the subbasal corneal nerve plexus in the central cornea and langerhans cells. Activated Langerhans cells were identified according to established morphological criteria: they presented with bright, highly reflective cell bodies of irregular or dendritic morphology, with visible dendritic processes extending from the soma. In contrast, non-activated Langerhans cells typically appeared as small, round structures with uniformly low reflectivity and no visible dendrites.

### 2.5 Hematoxylin–eosin staining

On day 35 of the experiment, guinea pigs were anesthetized with 3% isofluranethrough inhalation and then sacrificed by neck removal, the lacrymal glands, corneas and trigeminal nerve roots were harvested from the guinea pigs. The tissues were fixed with 2% paraformaldehyde, followed by dehydration, clearing, paraffin infiltration, and embedding. After the paraffin had completely solidified, sections were cut. Sections of the lacrimal gland and cornea were cut at a thickness of 5 μm, and sections of the trigeminal ganglion at 10 μm. Corneal sections were obtained from the central cornea, while sections of the lacrimal gland and trigeminal ganglion were harvested from the central region of each tissue block. Three non-consecutive sections were selected from each tissue per animal for quantitative analysis, and three random fields of view were analyzed per section. The paraffin sections were placed on a 39 °C water bath to enable natural stretching, then mounted on glass slides and baked for 30–60 min. Finally, staining and imaging were performed.

### 2.6 Transmission electron microscopy

The trigeminal nerve roots were pre-fixed in 2% paraformaldehyde with 2.5% glutaraldehyde and post-fixed with 1% osmium tetroxide buffer solution, followed by dehydration, resin infiltration, and polymerization embedding. Semi-thin sections (0.8 μm thick) of the embedded blocks were cut, stained with toluidine blue, and observed under a light microscope for localization. Areas with dense myelin sheaths were selected for ultra-thin sectioning (90 nm thick), which were subsequently mounted on 200-mesh copper grids with Formvar films. After routine uranyl acetate-lead citrate electron staining and air-drying, imaging was performed.

### 2.7 Neural tracing

#### 2.7.1 Single tracing

Guinea pigs were anesthetized with 3% isoflurane via inhalation and fixed. A microinjector (Hamilton, United States) was used to inject 6 μL of 1% CTB-488 (Invitrogen-Molecular Probes, Thermo Fisher Scientific, United States) into the left periocular acupoints (BL1, BL2, SJ23, GB1, and EX-HN5) and non-acupoint areas (N1, N2, N3, N4, and N5) at a depth of 3 mm. The needle was retained for 1 min after injection.

#### 2.7.2 Double tracing

Guinea pigs were anesthetized with 3% isoflurane via inhalation and fixed with a stereotaxic instrument. A 1.5-cm incision was made along the midline of the skull, and the subcutaneous tissues were separated. A microinjector was fixed on the stereotaxic instrument, with the needle tip aligned with the Bregma point as the zero point. The needle was moved 3.14 mm posteriorly and 3.2 mm leftward, and the injection site was marked with a marker pen. The microinjector was removed, and a 2-mm hole was drilled. The dura mater was incised, and the microinjector was filled with 4% FG (Fluorochrome, United States). The needle tip was lowered to the brain surface, then rapidly lowered by 7.0 mm and retracted by 0.2 mm (i.e., the injection depth was 6.8 mm) (30). FG (0.2 μL) was injected at a rate of 0.2 μL/min, and the needle was retained for 5 min before being raised above the brain surface and withdrawn. The skin on both sides was sutured (31, 32). Meanwhile, 6 μL of 1% CTB-488 was injected into the left periocular acupoints [JingMing (BL1), CuanZhu (BL2), SiZhukong (SJ23), TongZiliao (GB1), and TaiYang (EX-HN5)] using a microinjector.

#### 2.7.3 Immunofluorescence technique

Five days after tracer injection, guinea pigs were anesthetized with 3% isoflurane via inhalation, the brainstem and trigeminal nerve roots were harvested following transcardial perfusion fixation. The tissues were immersed in a 25% sucrose solution for 24 h for dehydration. Tissue segments (2–3 mm in length) of the dehydrated brainstem and trigeminal nerve roots were excised, embedded in OCT freezing medium, and frozen. Serial sections of 30 μm thick brainstem specimens and 20 μm thick trigeminal nerve root specimens (10 sections each) were prepared using a cryostat and directly attached to glass slides. Images were observed and collected using upright fluorescence and confocal microscopes, respectively, and positive neurons were counted using ImageJ software.

### 2.8 Electrophysiology

Guinea pigs were anesthetized and euthanized with 3% isoflurane through inhalation. Their brains were rapidly extracted, and 250 μm coronal sections were prepared using a vibratome (Leica VT1200S) in cold (0–4 °C) sucrose-based artificial cerebrospinal fluid (SB-ACSF) containing (in mM): 40 NaCl, 148.5 sucrose, 4 KCl, 1.25 NaH₂PO₄, 25 NaHCO₃, 0.5 CaCl₂, 7 MgCl₂, 10 glucose, 1 sodium ascorbate, 3 sodium pyruvate, and 3 myoinositol, saturated with 95% O₂ and 5% CO₂. After recovery for 45 min at 32 °C in an oxygenated (95% O₂/5% CO₂) 1:1 mixture of SB-ACSF and ACSF, slices were kept in the same medium at room temperature for the rest of the day and individually transferred to a recording chamber continuously perfused at 2–3 mL/min with oxygenated ACSF. The ACSF contained the following (in mM): 125 NaCl, 4.5 KCl, 2.5 CaCl₂, 1.3 MgCl₂, 1.25 NaH₂PO₄, 25 NaHCO₃, 15 sucrose, and 15 glucose. Patch pipettes (5–8 MΩ) were pulled from thin-walled borosilicate glass using a micropipette puller (Sutter Instrument, Novato, CA, United States). Neurons were visualized using an upright microscope with an IR-DIC lens and illuminated with a white light source (Nikon, Japan). Whole-cell patch-clamp and cell-attached recordings were made using an IPA-2 integrated patch amplifier controlled with SutterPatch software (Sutter Instrument, Novato, CA, United States). The resting membrane potential (RMP) of all neurons was first recorded and subsequently adjusted to −70 mV for electrophysiological assessments of excitability. Action potential firing was induced by a series of 300 ms current steps (−60 pA to +500 pA) incremented in +40 pA steps. Recordings were analyzed offline for the number of spikes in response to each current step, threshold (mV), rheobase (pA), action potential peak amplitude (mV), and action potential half-width (ms) using Clamfit software (33, 34).

### 2.9 Statistical analysis

All data were analyzed and presented using GraphPad Prism 10.1.2. Data were expressed as mean ± standard error of the mean (SEM). Data normality was assessed using the Shapiro–Wilk test prior to statistical analysis. For comparisons among multiple groups, one-way or two-way analysis of variance (ANOVA) was applied as appropriate. Comparisons between two groups were performed using the *t*-test. For two-way ANOVA, when significant main effects or interactions were detected, Tukey’s multiple comparisons test was used for post-hoc analysis of normally distributed data. Statistical significance was set at *P* < 0.05.

## 3 Results

### 3.1 Periocular acupuncture improves ocular surface abnormalities and sensory function in dry

**Figure 2:**
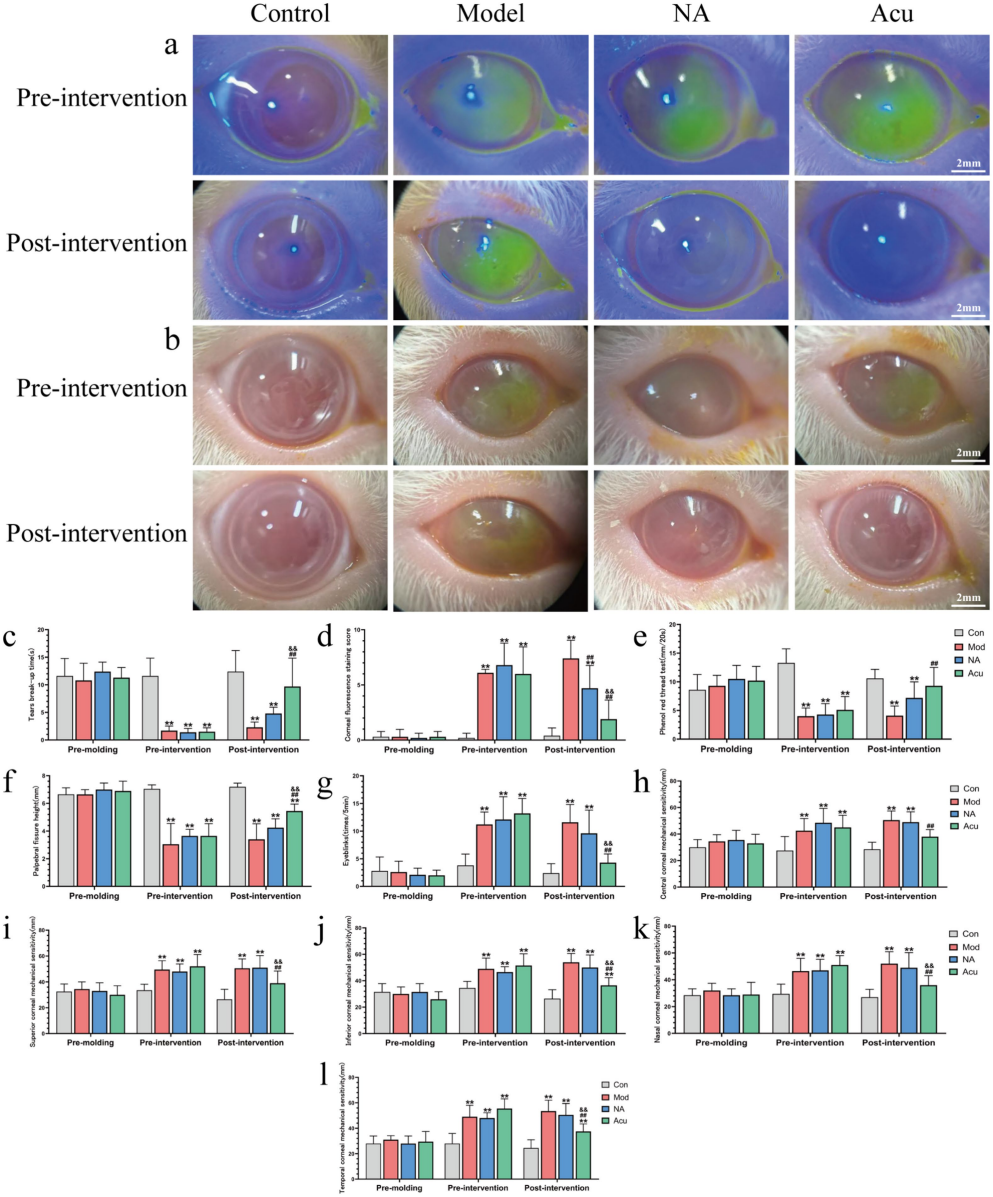
Periocular acupuncture improves ocular surface abnormalities and sensory function in dry eye. **(a)** Slit-lamp images of corneal fluorescein staining in guinea pigs pre- and post-intervention (scale bar = 2 mm). **(b)** Palpebral fissure height images of guinea pigs pre- and post-intervention. (scale bar = 2 mm). **(c)** Tear film break-up time. **(d)** Corneal fluorescein staining scores. **(e)** Phenol red thread test results. **(f)** Palpebral fissure height. **(g)** Blinking frequency within 5 min. **(h)** Central corneal sensation. **(i)** Superior corneal sensation. **(j)** Inferior corneal sensation. **(k)** Nasal corneal sensation. **(l)** Temporal corneal sensation (*n* = 10, binocular measurements, both eyes were treated as independent samples. measurement times: days 0, 21, and 35. Compared with the Con group, ^**^P < 0.01; compared with the Mod group, ^##^*P* < 0.01; compared with the NA group, ^&&^*P* < 0.01).

Eye acupuncture effectively improved ocular surface abnormalities and sensory function. Dry eye parameter assessments showed that compared with the Mod group, periocular acupuncture significantly prolonged tear film break-up time (2.30 ± 1.92 vs. 9.70 ± 5.14 s, Figure 2c, *P* < 0.01), reduced corneal fluorescein staining (7.40 ± 3.31 vs. 1.90 ± 1.73 points, Figures 2a,d, *P* < 0.01), and increased tear secretion (4.10 ± 3.06 vs. 9.30 ± 3.20 mm/20 s, Figure 2e, *P* < 0.01). The NA group also showed a certain improvement in the above parameters, however, significant differences compared were observed with periocular acupuncture (4.80 ± 1.14 vs. 9.70 ± 5.14 s; 4.70 ± 2.06 vs. 1.90 ± 1.73 points; 7.20 ± 2.78 vs. 9.30 ± 3.20 mm/20 s, Figures 2c–e, *P* < 0.01). Ocular surface sensory assessments demonstrated that compared with the Mod group, periocular acupuncture significantly increased palpebral fissure height (3.40 ± 2.30 vs. 5.45 ± 0.50 mm, Figures 2b,f, *P* < 0.01) and decreased blinking frequency (11.60 ± 6.60 vs. 4.30 ± 1.57 times/5 min, Figure 2g, *P* < 0.01). However, acupuncture at non-acupoint areas did not significantly improve ocular surface sensory abnormalities, and no statistical differences were observed in palpebral fissure height and blinking frequency compared with the Mod group (4.25 ± 0.63 vs. 3.40 ± 2.30 mm; 9.60 ± 4.20 vs. 11.60 ± 6.60 times/5 min, Figures 2f,g, *P* > 0.05). Regarding corneal sensation, compared with the Con group, the Mod group demonstrated significantly increased corneal sensation in all regions (central, superior, inferior, nasal, and temporal) (28.50 ± 5.30 vs. 50.50 ± 13.82 mm, 26.50 ± 7.84 vs. 50.50 ± 14.65 mm, 26.50 ± 6.69 vs. 54.00 ± 13.20 mm, 27.00 ± 5.87 vs. 52.00 ± 17.17 mm, 24.50 ± 6.43 vs. 53.50 ± 16.97 mm, Figures 2h–l, *P* < 0.01). Compared with the Mod group, the Acu group showed significantly decreased corneal sensation in all regions, approaching normal levels (50.50 ± 13.82 vs. 38.00 ± 5.37 mm, 50.50 ± 14.65 vs. 39.00 ± 9.37 mm, 54.00 ± 13.20 vs. 36.50 ± 5.80 mm, 52.00 ± 17.17 vs. 36.00 ± 6.99 mm, 53.50 ± 16.97 vs. 37.50 ± 5.89 mm, Figures 2h–l, *P* < 0.01), while the NA group showed no statistical differences in corneal sensation in all regions compared with the Mod group (50.50 ± 13.82 vs. 49.00 ± 7.75 mm, 50.50 ± 14.65 vs. 51.00 ± 9.37 mm, 54.00 ± 13.20 vs. 50.00 ± 9.43 mm, 52.00 ± 17.17 vs.49.00 ± 11.25 mm, 53.50 ± 16.97 vs. 50.50 ± 8.96 mm, Figures 2h–l, *P* > 0.05). Additionally, we found that with increased modeling time, the corneal sensation in all regions of the Mod group showed a rising trend, indicating that long-term dry eye status may enhance corneal sensitivity.

### 3.2 Acupuncture exerts significant improvement effects on the structure and histology of the cornea, lacrimal glands, and TG in dry eye model guinea pigs

**Figure 3:**
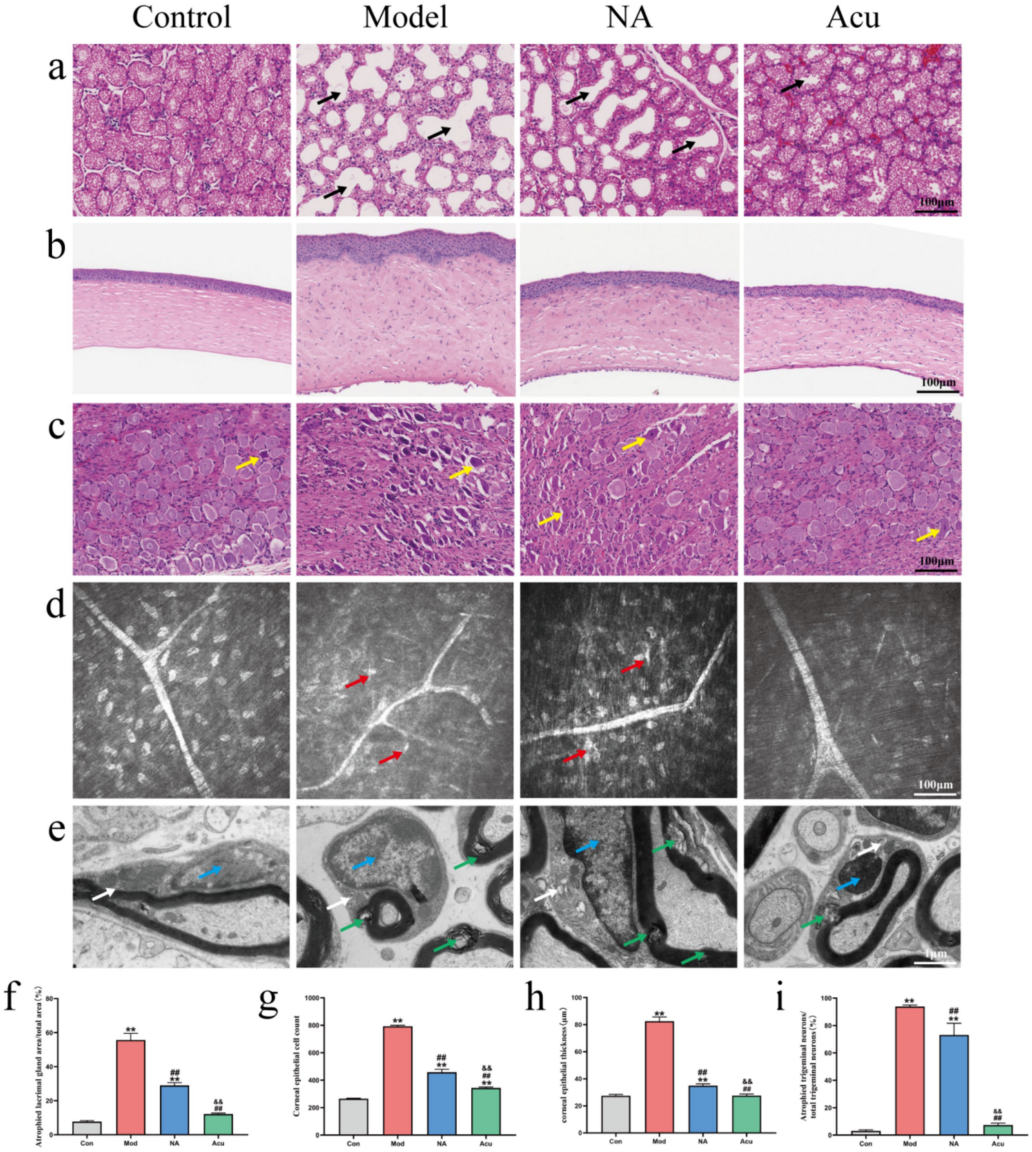
Improvement effects of acupuncture on the structure and histology of the cornea, lacrimal glands, and trigeminal ganglia in dry eye model guinea pigs. **(a)** HE staining of lacrimal gland cross-sections in each group (Black arrow: atrophic lacrimal gland vacuolesscale, bar = 100 μm). **(b)** HE staining of corneal cross-sections in each group (scale bar = 100 μm). **(c)** HE staining of TG cross-sections in each group (Yellow arrow: atrophic trigeminal neurons, scale bar = 100 μm). **(d)** *In vivo* confocal microscopy images of the subbasal corneal nerve plexus in guinea pigs (Red arrow: activated Langerhans cells, scale bar = 100 μm). **(e)** Transmission electron microscopy images of TG in each group (Blue arrow: Schwann cell nucleus; Green arrow: loosened myelin sheath; White arrow: rough endoplasmic reticulum, scale bar = 1 μm). **(f)** Proportion of lacrimal gland atrophy area to total area (%). **(g)** Number of corneal epithelial cells in guinea pigs. **(h)** Corneal epithelial thickness (μm). **(i)** Proportion of atrophic TG neurons to total TG neurons (%) (*n* = 3; compared with the Con group, ^**^P < 0.01; compared with the Mod group, ^##^*P* < 0.01; compared with the NA group, ^&&^*P* < 0.01).

Based on the previous observation that acupuncture improves ocular surface and sensory function, we further observed its effects on lacrimal gland and corneal histopathology. Meanwhile, since dry eye is associated with sensory nerve abnormalities and the trigeminal nerve is the main nerve responsible for ocular surface sensation, we also explored the effect of acupuncture on the histopathology of the trigeminal sensory nerve. HE staining of the lacrimal glands demonstrated that compared with the Con group, the Mod group had obvious atrophy of lacrimal gland epithelial cells, dilated glandular lumens, reduced secretion, and significantly increased lacrimal gland atrophy area (7.70 ± 0.60 vs. 51.65 ± 3.60%, Figures 3a,f, *P* < 0.01). Compared with the Mod group, the Acu and NA groups exhibited improved atrophy of lacrimal gland epithelial cells; however, some glandular lumens remained dilated, and the proportion of lacrimal gland atrophy area was decreased (51.65 ± 3.60 vs. 12.16 ± 0.56%, 51.65 ± 3.60 vs.28.98 ± 1.56%, Figures 3a,f, *P* < 0.01). Meanwhile, the Acu group demonstrated a significantly better improvement in lacrimal gland atrophy than the NA group (12.16 ± 0.56 vs. 28.98 ± 1.56%, Figures 3a,f, *P* < 0.01). HE staining of the cornea revealed that, compared with the Con group, the Mod group had a thickened corneal epithelial layer, a significantly increased number of epithelial cells (27.48 ± 1.02 vs. 85.58 ± 3.09 μm; 265.33 ± 3.21 vs. 793.33 ± 6.84 cells, Figures 3b,g,h, *P* < 0.01), disorganized arrangement of squamous epithelial cells and stromal fibers, massive inflammatory cell infiltration, wrinkling of the Bowman’s and Descemet’s membranes, and extensive loss of endothelial cells. The Acu group showed regular arrangement of corneal epithelial cells (Figure 3b). Compared with the Mod group, both the Acu and NA groups had a significantly decreased number of epithelial cells (85.58 ± 3.09 vs. 27.62 ± 1.09 μm, 85.58 ± 3.09 vs. 34.90 ± 1.37 μm; 793.33 ± 6.84 vs. 343.67 ± 6.66 cells, 793.33 ± 6.84 vs. 458.00 ± 21.63 cells, Figures 3b,g,h, *P* < 0.01), with the Acu group showing a significantly better effect than the NA group (27.62 ± 1.09 vs. 34.90 ± 1.37 μm; 343.67 ± 6.66 vs. 458.00 ± 21.63 cells, Figures 3b,g,h, *P* < 0.01). The above results indicate that acupuncture can alleviate corneal and lacrimal gland tissue damage caused by dry eye, which is consistent with the improvements in corneal fluorescein staining and tear secretion observed in our study. Subsequently, we further observed the tissues that cause changes in ocular surface sensory function. HE staining of the TG showed that compared with the Con group, the Mod group had atrophic TG neurons, pyknotic nuclei, irregular arrangement, discontinuous and disorganized tissue structure, and a significantly increased percentage of atrophic nerve cells (3.07 ± 0.77 vs. 93.87 ± 1.13%, Figures 3c,i, *P* < 0.01). Compared with the Mod group, both the Acu and NA groups had a significantly decreased percentage of atrophic neurons (93.87 ± 1.13 vs. 7.34 ± 1.40%, 93.87 ± 1.13 vs. 73.16 ± 8.48%, Figures 3d,i, *P* < 0.01), and the Acu group showed a significantly better improvement in TG neuron atrophy than the NA group (7.34 ± 1.40 vs. 73.16 ± 8.48%, Figures 3d,i, *P* < 0.01). IVCM of the cornea revealed that compared with the Con group, the Mod group had tortuous and deformed subbasal corneal nerve plexus, changed width, decreased reflectivity of nerve branches with intermittent branches, and a large number of activated Langerhans cells. The NA group showed limited improvement in nerve structure, while periocular acupuncture significantly ameliorated these abnormalities (Figure 3d). TEM revealed that compared with the Con group, the Mod group had mild abnormalities in the ultrastructure of Schwann cells, including local loosening and disorganization of myelin sheaths in multiple areas, and dilated cisternae of rough endoplasmic reticulum with moderate electron-dense contents. The NA group still showed multiple areas of myelin sheath loosening and disorganization, whereas the Acu group had reduced abnormalities in the ultrastructure of Schwann cells, decreased myelin sheath loosening and disorganization, and normal rough endoplasmic reticulum (Figure 3e). The above results indicate that acupuncture can reverse the pathological disorder of corneal tissue and lacrimal gland atrophy in dry eye model guinea pigs, improve the structural abnormalities of the subbasal corneal nerve plexus, and alleviate the pathological damage of TG and the ultrastructural disorder of Schwann cells. Therefore, we speculate that the therapeutic effect of periocular acupuncture on dry eye neuropathic pain may be achieved by regulating the nerve conduction pathway of the trigeminal nerve.

### 3.3 Labeled neurons observed in the TG and VPM brain regions after single periocular acupoint injection of CTB-488

**Figure 4:**
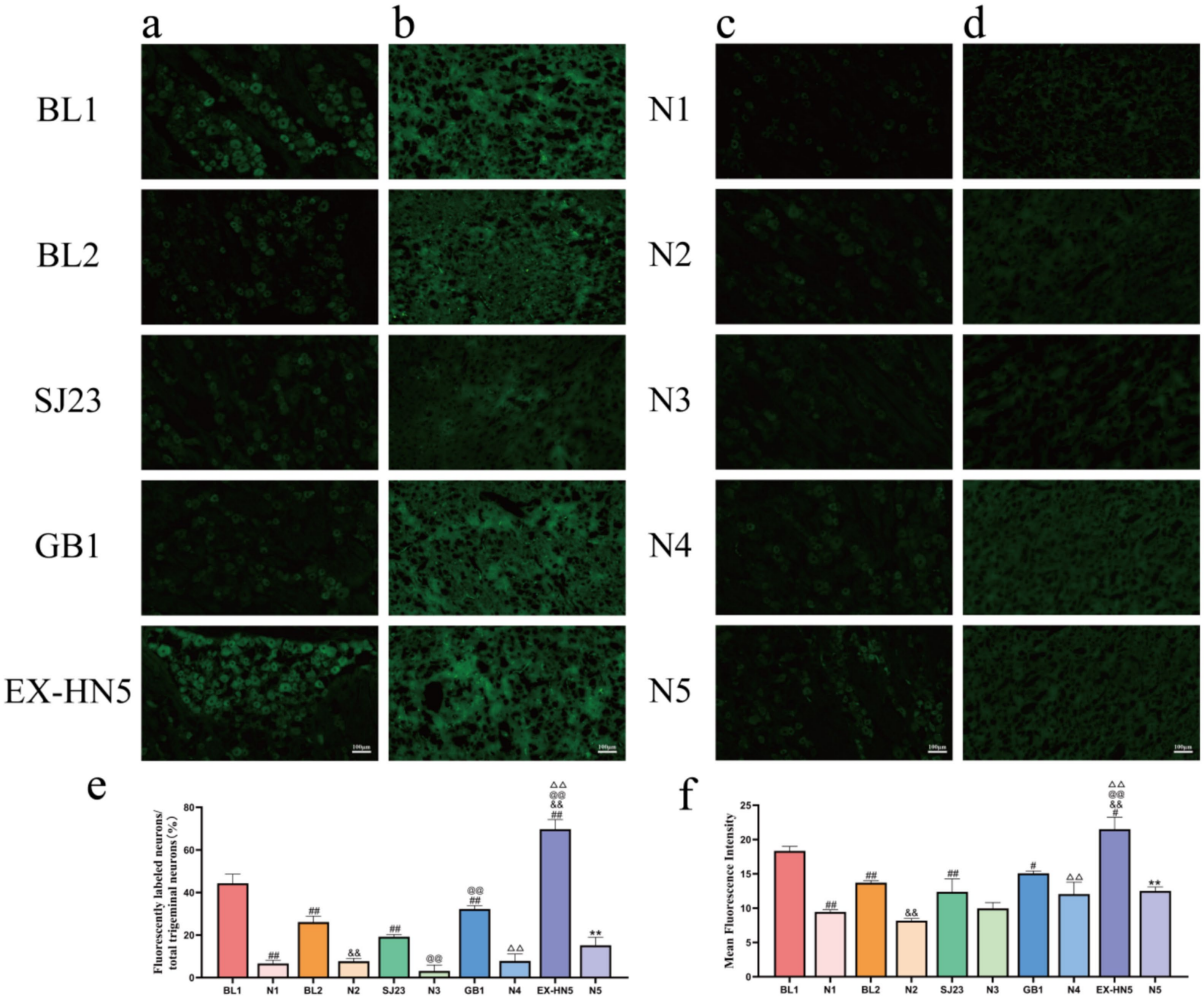
Neural staining in the TG and VPM brain regions after a single periocular acupoint injection of CTB-488. **(a)** Fluorescence microscope images of the left TG in the single periocular acupoint group. **(b)** Fluorescence microscope images of the right VPM in the single periocular acupoint group. **(c)** Fluorescence microscope images of the left TG in the single periocular non-acupoint group. **(d)** Fluorescence microscope images of the right VPM in the single periocular non-acupoint group. **(e)** Proportion of CTB-488-labeled neurons to total TG neurons in the left TG of the periocular acupoint and non-acupoint groups (%, *n* = 3). **(f)** Average fluorescence intensity of neurons in the right VPM region of the periocular acupoint and non-acupoint groups (*n* = 3) (scale bar = 100 μm. Compared with BL1, ^#^*P* < 0.05, ^##^*P* < 0.01; compared with BL2, ^&&^*P* < 0.01; compared with SJ23, ^@@^*P* < 0.01; compared with GB1, ^△△^*P* < 0.01; compared with EX-HN5, ^**^P < 0.01. The non-acupoint group was only compared with the corresponding acupoint group for differences).

To examine whether periocular acupuncture regulates the trigeminal nerve conduction pathway, we assessed the effect of single periocular acupoints on the trigeminal nerve conduction pathway. We injected CTB-488 peripherally into the left JingMing (BL1), CuanZhu (BL2), SiZhukong (SJ23), TongZiliao (GB1), and TaiYang (EX-HN5) acupoints in different guinea pigs, and simultaneously injected CTB-488 into the non-acupoint areas 0.5 cm away from each left acupoint to observe the neuronal expression in the trigeminal nerve pathway: TG–VPM. CTB-488-labeled neurons were located in the left (ipsilateral) TG and right (contralateral) VPM (Figures 4a–d). After injecting CTB-488 into the periocular acupoints (BL1, BL2, SJ23, GB1, and EX-HN5) and non-acupoint areas (N1–N5), labeled neurons were observed in the TG (Figures 4a,c). However, the number of labeled neurons in the acupoint group was significantly higher than that in the non-acupoint areas, and differences were observed among the acupoints. The number of labeled neurons in EX-HN5 (69.74 ± 4.49%) and BL1 (44.34 ± 4.32%) was significantly higher than that in GB1 (32.23 ± 1.59%), BL2 (26.11 ± 2.76%), and SJ23 (19.27 ± 0.95%) (Figure 4e, *P* < 0.01), indicating that the targeting of periocular acupoints to the peripheral sensory TG has regional specificity. In the VPM, the tracer fluorescence intensity corresponding to the Acu group was also significantly higher than that in the NA group, and showed a consistent correlation with the specific differences of acupoints in the TG. The tracer fluorescence intensity in EX-HN5 (21.50 ± 1.76 a.u.) and BL1 (17.69 ± 0.83 a.u.) was significantly higher than that in GB1 (16.42 ± 1.27 a.u.), BL2 (13.71 ± 0.32 a.u.), and SJ23 (12.382 ± 1.88 a.u.) (Figure 4f, *P* < 0.05). These results indicate that the periocular acupoints EX-HN5, BL1, GB1, BL2, and SJ23 have specific neural pathways targeting the “peripheral (TG)–central (VPM)” level, with significantly higher labeling efficiency on the peripheral sensory ganglion than on the non-acupoint areas, and there are targeting differences among the acupoints.

### 3.4 Labeled neurons observed in the TG and VPM nuclei after central and peripheral double tracing

**Figure 5:**
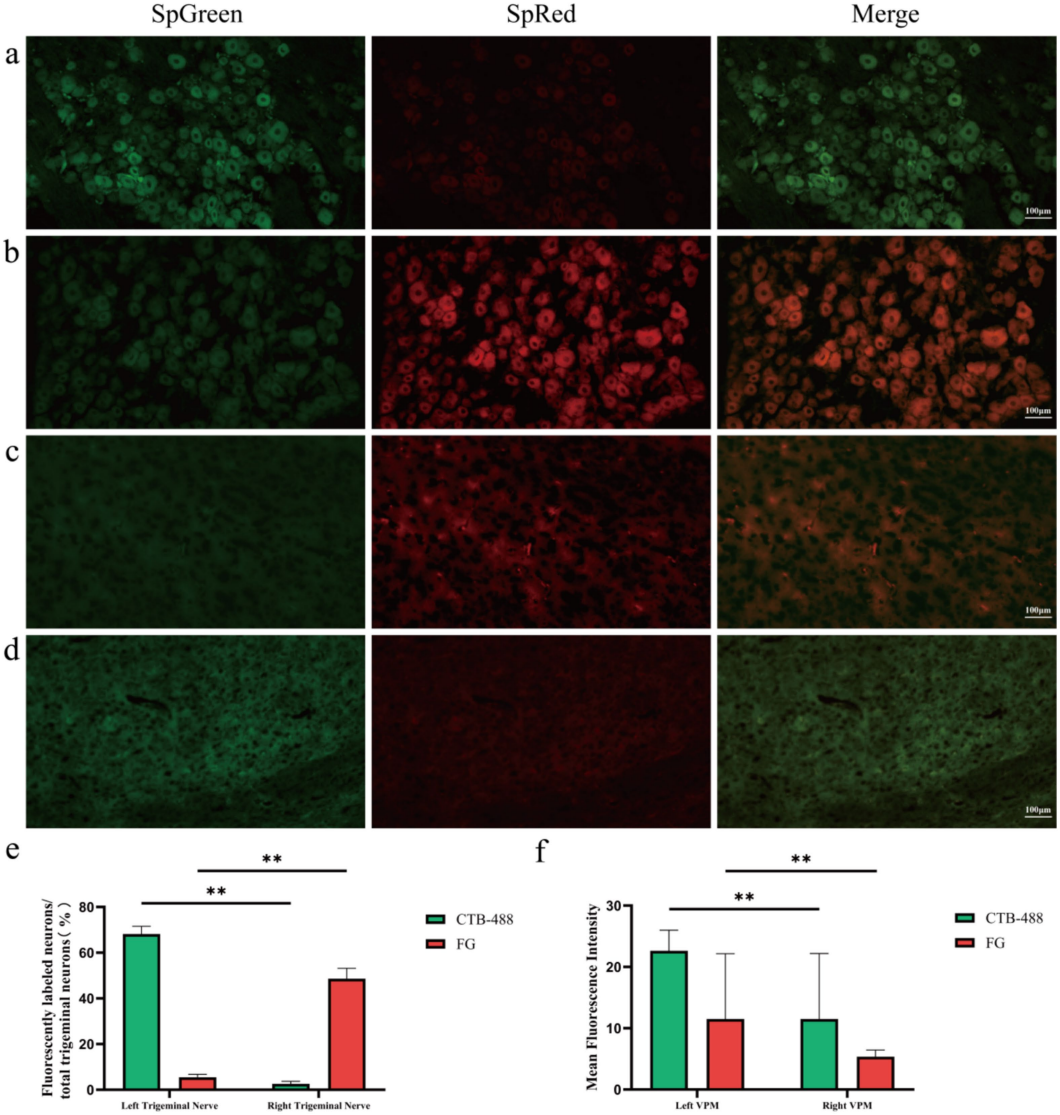
Labeled neurons observed in the TG and VPM nuclei after central and peripheral double tracing. **(a)** Fluorescence microscope images of the left TG. **(b)** Fluorescence microscope images of the right TG. **(c)** Fluorescence microscope images of the left VPM neurons. **(d)** Fluorescence microscope images of the right VPM neurons (scale bar = 100 μm, CTB-488 labeled green, FG labeled red). **(e)** Proportion of CTB-488 and FG-labeled neurons to total TG neurons in the TG (%, *n* = 3, ^**^P < 0.01). **(f)** Average fluorescence intensity of neurons in the VPM region (*n* = 3, ^**^P < 0.01).

To further confirm the dual signal conduction of periocular acupuncture on the “TG–VPM,” we used stereotaxic technology to inject FG centrally into the left VPM nucleus of the same guinea pig. Simultaneously, we injected CTB-488 peripherally into the left BL1, BL2, SJ23, GB1, and EX-HN5 acupoints to observe the dual neuronal expression in the “periocular acupoint–TG–VPM.” FG-labeled neurons (red) were located in the right (contralateral) TG and left (ipsilateral) VPM (Figures 5b,c), while the fluorescence-labeled neurons in the left (ipsilateral) TG and right (contralateral) VPM were not obvious (Figures 5a,d). Statistical analysis of FG-labeled TG neurons revealed that the proportion in the right TG (48.64 ± 4.52%) was significantly higher than that in the left TG (2.64 ± 1.03%) (Figure 5e, *P* < 0.01). Statistical analysis showed that the fluorescence intensity of FG-labeled neurons in the left VPM (24.62 ± 1.22 a.u.) was significantly higher than that in the right VPM (5.37 ± 1.07 a.u.) (Figure 5f, *P* < 0.01), indicating that the “peripheral (TG)-central (VPM)” conduction pathway is still valid for retrograde tracing of the VPM. After injecting CTB-488 into the left periocular acupoint group, labeled neurons (green) were observed in the left (ipsilateral) TG and right (contralateral) VPM (Figures 5a,d), whereas the labeled neurons in the right (contralateral) TG and left (ipsilateral) VPM were not obvious (Figures 5b,c). Additionally, statistical analysis revealed that the proportion of CTB-488-labeled neurons in the left TG (68.16 ± 3.38%) was significantly higher than that in the right TG (5.51 ± 1.27%) (Figure 5e, *P* < 0.01). The analysis also showed that the fluorescence intensity of CTB-488-labeled neurons in the left VPM (22.64 ± 3.35 a.u.) was significantly higher than that in the right VPM (5.53 ± 0.57 a.u.) (Figure 5f, *P* < 0.01), which is consistent with the results of single tracing. The results of anterograde and retrograde double tracing confirm the existence of a specific neural conduction pathway of “periocular acupoint–TG–VPM,” indicating that periocular acupoint stimulation can specifically project to the VPM via the TG.

### 3.5 Periocular acupuncture targets the VPM to regulate neuronal electrophysiological function

**Figure 6:**
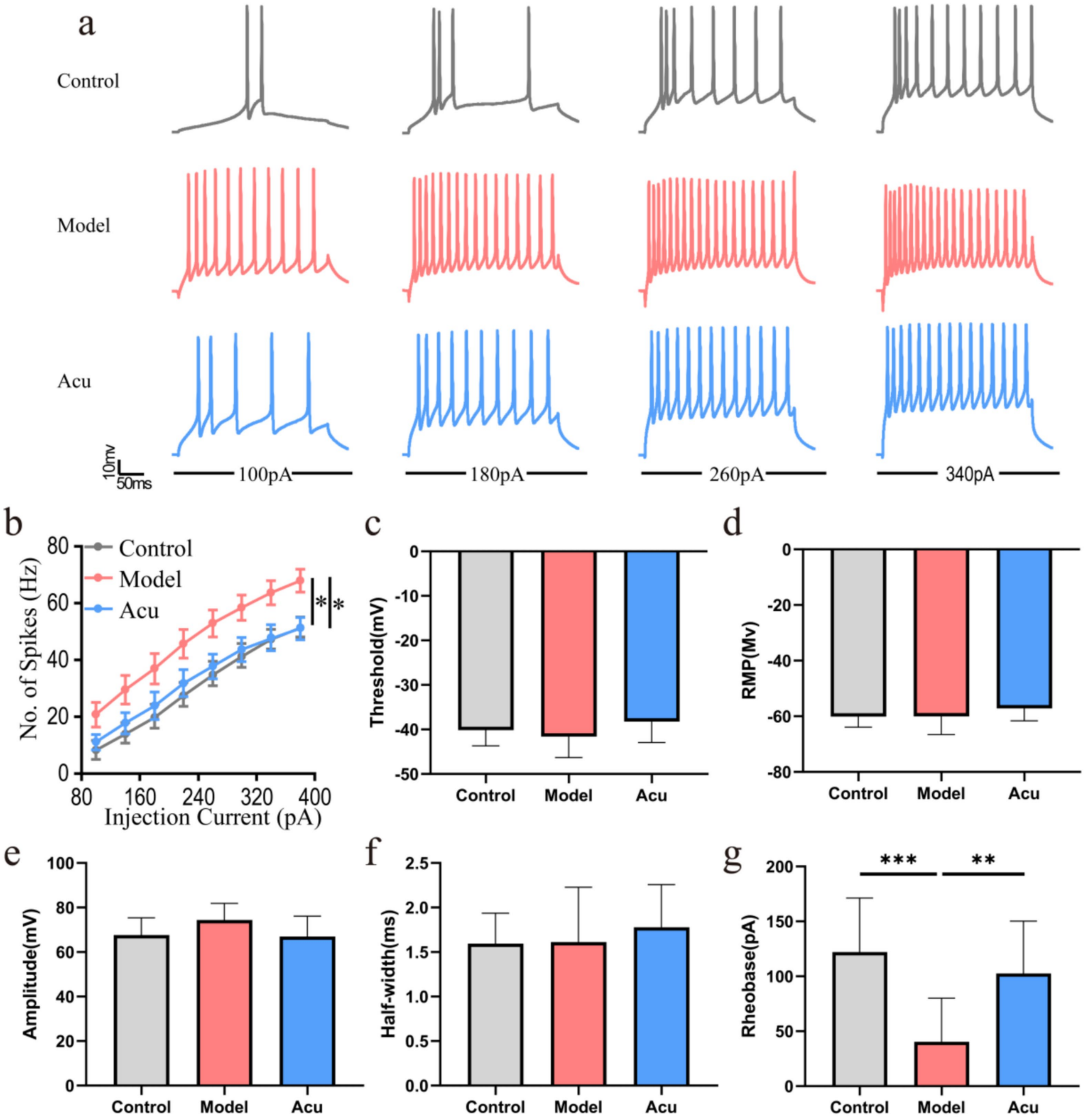
Periocular acupuncture targets the ventral posteromedial thalamic nucleus to regulate neuronal electrophysiological function. **(a)** Representative spikes of current-evoked firing in VPM neurons from the Control, Model, and Acu groups. **(b)** Step current injection induced a significant increase in the spike numbers of VPM neurons in the three groups of guinea pigs. **(c)** Bar graph indicating the distribution of individual AP thresholds measured in VPM neurons. **(d)** Bar graph showing the AP resting membrane potential measured in VPM neurons. **(e)** Bar graph showing the AP amplitude measured in VPM neurons (mV). **(f)** Bar graph displaying the AP half-widths measured in VPM neurons (ms). **(g)** Bar graph indicating the mean rheobase current in VPM neurons (pA) (*n* = 13 neurons, 3 guinea pigs; ^*^P < 0.05; ^**^P < 0.01; ^***^P < 0.001).

Combining the results from single CTB and double FG tracing, we further confirmed that the VPM nucleus neurons overlap with the neural pathways related to peripheral acupuncture, suggesting that the VPM is a key nucleus for periocular acupuncture-mediated peripheral–central neural regulation. Based on this pathway evidence, we used patch-clamp technology to assess the electrophysiological function of VPM neurons. We found no significant differences in the basic waveform of action potentials (threshold, amplitude, half-width, and RMP) of VPM neurons among the Mod, Con, and Acu groups (Figures 6c–f). The possible reason is that the neuronal function abnormalities in the Mod and Acu groups are only focused on the level of excitability (firing frequency and threshold), rather than the above basic electrophysiological parameters. Therefore, we detected the Spike and Rheobase values of each group under different action potentials, and found that the number of action potential firings (Spike) of VPM neurons in the Con group (2.82 ± 4.04–47.18 ± 11.85 Hz) was small under different injected current stimulations. In contrast, the Spike number of VPM neurons in the Mod group showed a significant upward trend with increasing injected current (100–340 pA), and the minimum current (Rheobase) triggering action potentials was significantly reduced (22.31 ± 15.72–68.97 ± 15.12 Hz; 40.48 ± 39.54 pA, Figures 6a,b,g, *P* < 0.05; *P* < 0.001), indicating that VPM neurons have functional abnormalities of hyperexcitability under pathological conditions. After periocular acupuncture treatment, the Spike number of VPM neurons was significantly reduced, and the Rheobase was increased (11.03 ± 9.66–51.03 ± 13.90 Hz; 102.56 ± 47.71 pA, Figures 6a,b,g, *P* < 0.05, *P* < 0.01), indicating that periocular acupuncture can inhibit the hyperexcitable state of neurons through the VPM nucleus.

## 4 Discussion

**Figure 7:**
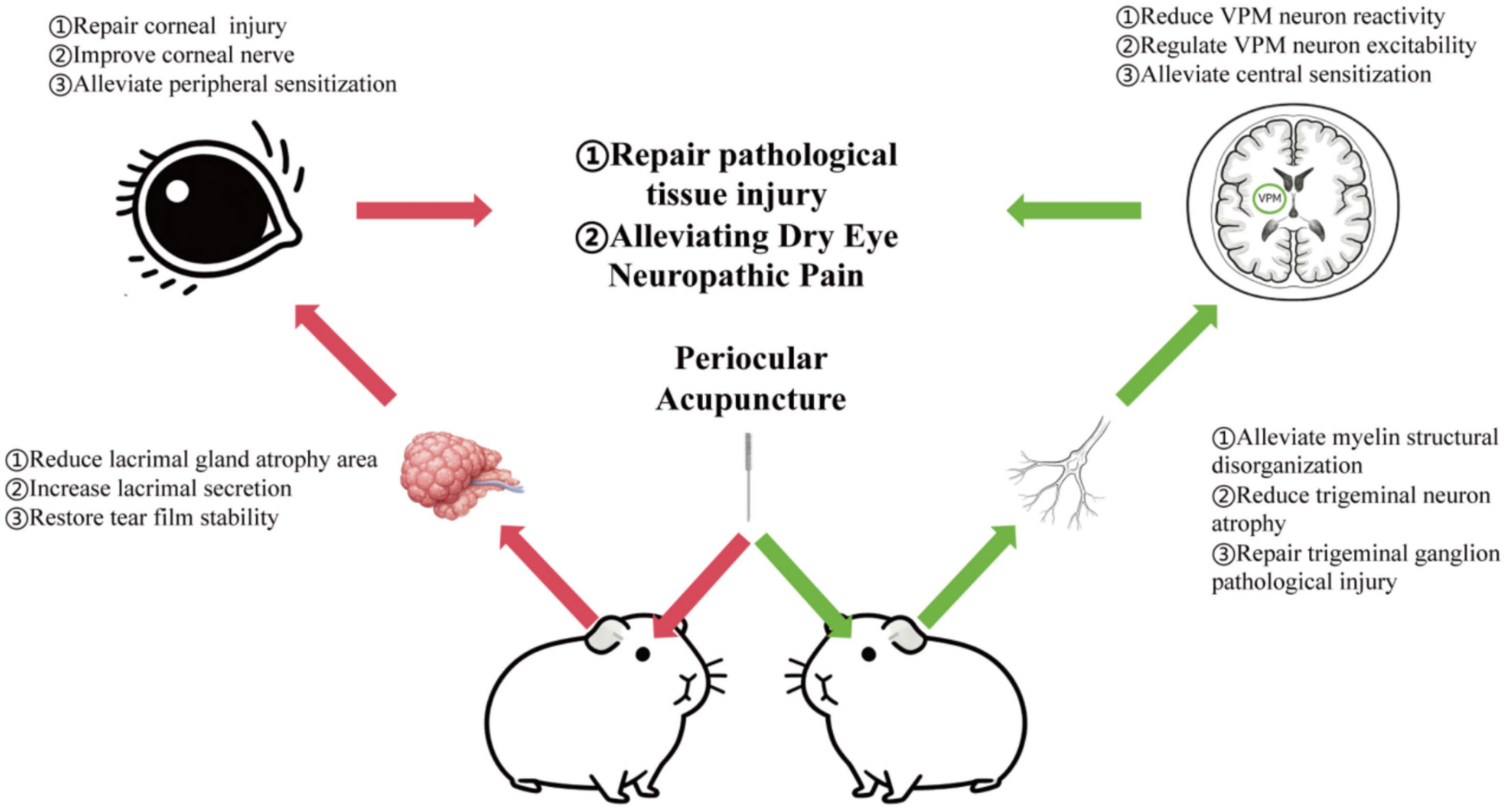
Peripheral and central mechanisms of acupuncture in the treatment of dry eye neuropathic pain.

DED is a multifactorial ocular surface disorder characterized by tear film instability, ocular surface inflammation, and sensory abnormalities. Among these, trigeminal nerve-mediated corneal hyperesthesia is a major factor severely affecting patients’ quality of life (35). As a complementary and alternative therapy, acupuncture has been clinically confirmed to alleviate dry eye symptoms (36, 37); however, its peripheral–central regulatory mechanism remains unclear. By integrating ocular surface function assessment, histopathological staining, neural tracing, and electrophysiological techniques, this study systematically explored the therapeutic effects and neurobiological mechanisms of periocular acupuncture on guinea pigs with dry eye neuropathic pain (Figure 7).

Scopolamine, an anticholinergic drug, induces dry eye by inhibiting parasympathetic nerve-mediated tear secretion, leading to reduced tear production. Long-term administration of scopolamine in animal models results in pathological characteristics that closely resemble clinical dry eye, including lacrimal gland atrophy, decreased tear secretion, corneal dryness, and epithelial damage (38). Benzalkonium chloride, a commonly used ophthalmic preservative, exhibits local toxic effects. It disrupts the corneal epithelial barrier, triggers inflammatory responses, and causes corneal tissue damage, thereby worsening the pathological conditions associated with dry eye. Additionally, benzalkonium chloride can activate corneal nerve endings, inducing or exacerbating corneal hyperalgesia. Studies have demonstrated that continuous administration of 0.2% benzalkonium chloride for 14 days significantly increases corneal fluorescein staining scores and leads to abnormalities in corneal nerve structure and nociceptive behaviors (39). Scopolamine-induced lacrimal gland dysfunction, when combined with benzalkonium chloride-induced corneal injury, more comprehensively simulates the multiple pathological features of clinical dry eye in patients with neuropathic pain. These include decreased tear secretion, ocular surface damage, abnormal corneal nerve function, and neuropathic pain. This model has been shown to stably maintain dry eye symptoms for at least 14 days, accompanied by measurable neurosensory abnormalities. Therefore, it is not only suitable for evaluating dry eye symptoms but also for studying the neuropathic pain associated with dry eye.

During model establishment, we observed that by day 10, due to the effects of benzalkonium chloride, guinea pigs exhibited severe corneal epithelial defects, edema, and even corneal ulceration, with corneal sensation nearly absent, making sensitivity measurements impossible. This was attributed to the severe early-stage corneal epithelial damage and edema induced by benzalkonium chloride, resulting in transient sensory abnormalities (40). At this stage, the ocular surface was in the acute injury phase and did not meet the criteria for a dry eye neuropathic pain model. Clinically, the onset and progression of dry eye neuropathic pain typically require a certain period of cumulative exposure. On day 21, we conducted a comprehensive evaluation of the ocular surface and sensory function. The results showed that corneal edema and ulceration had mostly resolved, and corneal morphology had returned to normal. However, corneal epithelial damage and reduced tear secretion persisted, and corneal sensation was significantly hypersensitive, indicating that the model state was stabilizing. Continuous modeling stimulation can stabilize corneal epithelial damage and induce a chronic abnormal state in neurosensory function (41). At this point, the model was successfully established and met the criteria for dry eye neuropathic pain. Therefore, day 22 was chosen as the starting time point for acupuncture intervention, a protocol that has been successfully applied in previous studies following 21 days of modeling. Day 35 was selected as the treatment endpoint, based on the need to observe the therapeutic effects of acupuncture. Acupuncture treatment typically requires a certain period to exert stable effects, especially in the context of neural structural repair and functional recovery. By comparing the results from day 22 and day 35, we can more objectively assess the cumulative and long-term regulatory effects of acupuncture on dry eye symptoms and neuropathic pain. Previous studies utilizing a “21-day modeling + 14-day intervention” protocol have successfully observed significant therapeutic effects of acupuncture in dry eye models (42). Therefore, day 35 as the treatment endpoint allows for a thorough evaluation of acupuncture’s regulatory effects on both the ocular surface and the nervous system, providing reliable data for subsequent mechanistic investigations.

This study’s results showed that periocular acupuncture can effectively improve the ocular surface function of guinea pigs with dry eye neuropathic pain, including reducing corneal fluorescein staining scores, prolonging tear film break-up time, increasing tear secretion, reducing abnormal blinking, restoring palpebral fissure height, and normalizing corneal sensation. Meanwhile, acupuncture can reverse the histopathological damage of the ocular surface and TG by significantly reducing the lacrimal gland atrophy area, repairing the morphological abnormalities of the subbasal corneal nerve plexus, reducing the activation of Langerhans cells in the cornea, and alleviating the pathological damage of TG neurons and the disorganization of Schwann cell myelin sheath structure. Notably, the therapeutic effect of periocular acupuncture is significantly better than that of acupuncture at non-acupoint areas, indicating that the stimulation of periocular acupoints has specificity, which is also supported by previous studies (43, 44). The improvement of the subbasal corneal nerve plexus and TG pathological state by periocular acupuncture is particularly critical. This experiment confirmed the pathological structural basis of “trigeminal nerve-mediated corneal hyperesthesia” in dry eye: corneal nerve damage induced by dry eye can trigger abnormal activation of sensory afferent signals, thereby aggravating ocular surface abnormalities and sensory disorders, whereas acupuncture-mediated repair of the trigeminal nerve structure can alleviate dry eye-related neuropathic pain.

Building on the confirmed therapeutic effects of acupuncture, neural tracing experiments further revealed periocular acupuncture’s anatomical basis. Notably, this study is the first to confirm the existence of a specific neural pathway of “periocular acupoint–TG–VPM” in a guinea pig model. The Acu group had a significantly higher number of labeled neurons and stronger fluorescence intensity in the TG and VPM than the NA group, confirming that periocular acupoints have a specific peripheral–central neural projection pathway. Additionally, the study revealed regional specificity of acupoints: the targeting ability of EX-HN5 and BL1 on the TG–VPM pathway is significantly stronger than that of GB1, BL2, and SJ23. BL1 is located at the medial canthus, where the supratrochlear nerve of the ophthalmic branch of the trigeminal nerve is distributed, which can directly stimulate the trigeminal nerve to produce positive signal feedback in the central nervous system through neural conduction (45); EX-HN5 is the convergence of the trigeminal nerve and ciliary ganglion, and acupuncture at EX-HN5 can directly stimulate the trigeminal nerve (46). The above results suggest that various periocular acupoints have different regulatory effects on the trigeminal nervous system. This finding is consistent with the clinical practice of “selecting specific acupoints for different diseases” and provides a morphological basis for optimizing acupoint compatibility in the clinical treatment of dry eye.

Furthermore, the NA group exhibited a certain degree of improvement in several ocular surface structural parameters, including corneal fluorescein staining, tear film break-up time and tear secretion volume, yet the extent of such improvement was significantly lower than that of the Acu group, which may arise from local microcirculatory enhancement, mechanical tissue responses or neural activation (47). However, in key sensory function indicators such as palpebral fissure height, blink frequency and corneal sensitivity, the NA group showed no significant improvement compared with the Mod group, whereas the Acu group achieved a marked amelioration of these indicators. In addition, neural tracing results revealed that tracer injection in non-acupoint areas resulted in a significantly lower number of labeled neurons in the trigeminal ganglion (TG) and ventral posteromedial thalamic nucleus (VPM) than in the Acu group, suggesting that the NA group failed to effectively activate the “periocular acupoint–TG–VPM” pathway. Therefore, although acupuncture at non-acupoints can ameliorate ocular surface structural damage to a certain extent, the alleviation of dry eye neuropathic pain—particularly the improvement of abnormal sensory perception and the reversal of central neuronal hyperexcitability—depends on the specific activation of the “periocular acupoint–TG–VPM” pathway by precise acupoint stimulation (48). This distinction also underscores the importance of acupoint specificity in the treatment of neuropathic pain conditions, where central sensitization represents the core pathological mechanism.

The electrophysiological analysis of VPM neurons further clarified the central regulatory mechanism of periocular acupuncture: in this study, no significant differences were observed in the basic electrophysiological parameters (action potential threshold, amplitude, half-width, and RMP) of VPM neurons among the Mod, Con, and Acu groups. However, the neuronal excitability indicators (Spike and Rheobase values) showed clear abnormalities and regulatory effects, indicating that this neuronal excitability is not caused by changes in the inherent properties of neurons themselves, but is related to the changes in central neuronal reactivity and plasticity induced by long-term chronic stimulation, which is consistent with the “central sensitization” characteristic of chronic pain (49, 50). After increased central neuronal reactivity, low-threshold, harmless stimuli are perceived as pain sensations with a larger range, longer duration, and higher intensity. Inflammation and injury are important factors triggering and maintaining increased neuronal reactivity, and a long-term increase in neuronal reactivity ultimately leads to central sensitization (51). During injury and disease progression, in response to these changes, the plasticity of the central and peripheral nervous systems undergoes adaptive changes (52). Periocular acupuncture exerts a targeted regulatory effect by inhibiting the hyperexcitability of VPM neurons and suppressing the transmission of abnormal sensory signals. We speculate that this regulation may involve modulating synaptic plasticity-related molecules (including NMDA receptors) or the function of specific ion channels (such as the TRP channel family) (53), which should be further verified at the molecular level in subsequent studies.

This study had some limitations. First, only male guinea pigs were used in the experiment, and the important factor of gender difference in pain and analgesia research was not considered (54). Second, *in vitro* brain slice electrophysiological recordings cannot fully simulate the complex neural network activities in the *in vivo* state. Third, this study only conducted tracing research on the specific conduction pathway from the peripheral to the central nervous system, and the conduction pathway from the central to the peripheral nervous system remains to be explored. Fourth, this study focused on the structural and functional changes of the trigeminal nerve pathway; however, the molecular mechanisms behind acupuncture regulation, including the roles of neurotransmitters, ion channels, and inflammatory factors, remain to be investigated.

## 5 Conclusion

This study provides evidence that periocular acupuncture can alleviate ocular surface dysfunction and trigeminal nerve-mediated dry eye neuropathic pain in guinea pig models with involvement of the specific neural pathway of “periocular acupoint–TG–VPM.” It reveals the pathological hyperexcitability of VPM neurons in the state of dry eye neuropathic pain and proves that periocular acupuncture can repair the pathological changes of the ocular surface and TG, inhibit the hyperexcitability of VPM neurons, and thereby block the amplification of abnormal pain sensory signals. This study not only provides a neurobiological basis for the clinical application of acupuncture in the treatment of dry eye neuropathic pain but also lays a foundation for further investigation into its molecular mechanisms and strategies to optimize clinical treatment.

## Data Availability

The raw data supporting the conclusions of this article will be made available by the authors, without undue reservation.
