# Supplementary material for: Mechanism of periocular acupuncture in alleviating dry eye neuropathic pain via regulation of the “periocular acupoint–trigeminal ganglion–ventral posteromedial thalamic nucleus” pathway
Source: Front Med (Lausanne). 2026 May 5;13:1803621. doi: 10.3389/fmed.2026.1803621 (PMC13183822; doi:10.3389/fmed.2026.1803621)
Supplement: Supplementary file 1 [file Data_Sheet_1.zip › Ethics approval/English translation.pdf]

Ethical Review Approval for Laboratory Animal Research at the Affiliated Hospital of Nanjing University of Chinese Medicine

Approval number	2024DW-038-02		
project name	Study on the Mechanism of Electroacupuncture Regulating NGF/TRPV1-mediated Dry Eye Neuralgia in Trigeminal Nerve Pathway		
Project source	Nanjing University of Chinese Medicine Horizontal		
Research unit	Jiangsu Provincial Hospital of Traditional Chinese Medicine		
Principal Investigator	Shen Huxing		
Review category	reexamine	Review method	Quick Review
Review date	24 April 2024	Review location	NA
juror	First trial fast-track : Zhou Jinyong Second trial fast-track : Zhou Jinyong		
audit-review file	1. Revised application form<br>2. Revised Research Protocol (Version No. : 4.0, Version Date : April 19,2024)		
Review comments			

After review by the Animal Ethics Committee, this study complies with the principles of animal protection, welfare, and ethics, and adheres to the relevant national regulations on laboratory animal welfare and ethics. The study is approved to proceed according to the approved protocol.

The study shall be conducted in accordance with the protocol approved by the ethics committee to ensure the welfare of animals.

If the principal investigator is changed during the study or any modifications are made to the research protocol, the applicant shall submit a revised application for review. In the event of any serious incident affecting the welfare of laboratory animals, the applicant shall promptly report to the ethics committee.

The applicant shall submit a progress report on the study at the frequency specified by the ethics committee. A written report must be submitted to the ethics committee one month prior to the deadline. If any situation arises that may significantly impact the experiment or increase animal risks, the applicant shall promptly submit a written report to the ethics committee. For projects

that exceed the validity period of the approval, fail to submit a progress report, and continue research without obtaining ethical review approval, the investigator must immediately cease all research activities, including interventions and data collection.<br>The applicant has requested to suspend or terminate the study early. Please submit the suspension/termination report promptly.<br>Upon completion of the study, the applicant shall submit a study completion report, as well as a summary report outlining the findings and conclusions of the study.	
Annual/Regular Review Frequency	Submit the research progress report one month before April 24,2025
term of validity	12 months
Contact and phone number	Zhao Xiaoqian 025-86560515
Signature of the President	
Ethics Committee	Ethical Committee for Laboratory Animals, Affiliated Hospital of Nanjing University of Chinese Medicine (Jiangsu Provincial Hospital of Traditional Chinese Medicine)
date	24 April 2024
