# Supplementary material for: Mechanism of periocular acupuncture in alleviating dry eye neuropathic pain via regulation of the “periocular acupoint–trigeminal ganglion–ventral posteromedial thalamic nucleus” pathway
Source: Front Med (Lausanne). 2026 May 5;13:1803621. doi: 10.3389/fmed.2026.1803621 (PMC13183822; doi:10.3389/fmed.2026.1803621)
Supplement: Supplementary file 1 [file Data_Sheet_1.zip › Ethics approval/Original document.pdf]

南京中医药大学附属医院实验动物研究
伦理审查批件

批件号	2024DW-038-02		
项目名称	电针调控三叉神经通路中 NGF/TRPV1 介导的干眼神经痛作用机制研究		
项目来源	南京中医药大学横向		
研究单位	江苏省中医院		
主要研究者	沈乎醒		
审查类别	复审	审查方式	快速审查
审查日期	2024 年 04 月 24 日	审查地点	NA
审查委员	初审快审：周锦勇　复审快审：周锦勇		
审查文件	1.修正的申请表<br>2.修正的研究方案（版本号：4.0，版本日期：2024 年 04 月 19 日）		

审查意见

经本实验动物伦理委员会审查，该研究符合动物保护、动物福利和伦理原则，符合国家实验动物福利伦理的相关规定，同意按所批准的方案开展本项研究。

请遵循伦理委员会批准的方案开展研究，保护动物的福利。

研究过程中若变更主要研究者，对研究方案有任何修改，请申请人提交修正案审查申请。

发生任何影响实验动物福利的严重事件，请申请人及时向伦理委员会提交报告。

请按照伦理委员会规定的定期审查频率，申请人在截止日期前 1 个月提交研究进展报告；当出现任何可能显著影响实验进行、或增加动物危险的情况时，请申请人及时向伦理委员会提交书面报告。超出批件有效期，没有提交研究进展报告并获得伦理审查批准继续研究的项目，研究者必须立即停止所有研究活动，包括干预措施和数据收集。

申请人暂停或提前终止研究，请及时提交暂停/终止研究报告。

完成研究，请申请人提交研究完成报告，以及概述研究发现和结论的总结报告。

年度/定期审查频率	请于 2025 年 04 月 24 日前 1 个月提交研究进展报告
有效期	12 个月
联系人与联系电话	赵晓倩　025-86560515
主席签字	
伦理委员会	南京中医药大学附属医院（江苏省中医院）实验动物伦理委员会（盖章）
日期	2024 年 04 月 24 日
