# Supplementary figures and images for: Mechanism of periocular acupuncture in alleviating dry eye neuropathic pain via regulation of the “periocular acupoint–trigeminal ganglion–ventral posteromedial thalamic nucleus” pathway
Source: Front Med (Lausanne). 2026 May 5;13:1803621. doi: 10.3389/fmed.2026.1803621 (PMC13183822; doi:10.3389/fmed.2026.1803621)

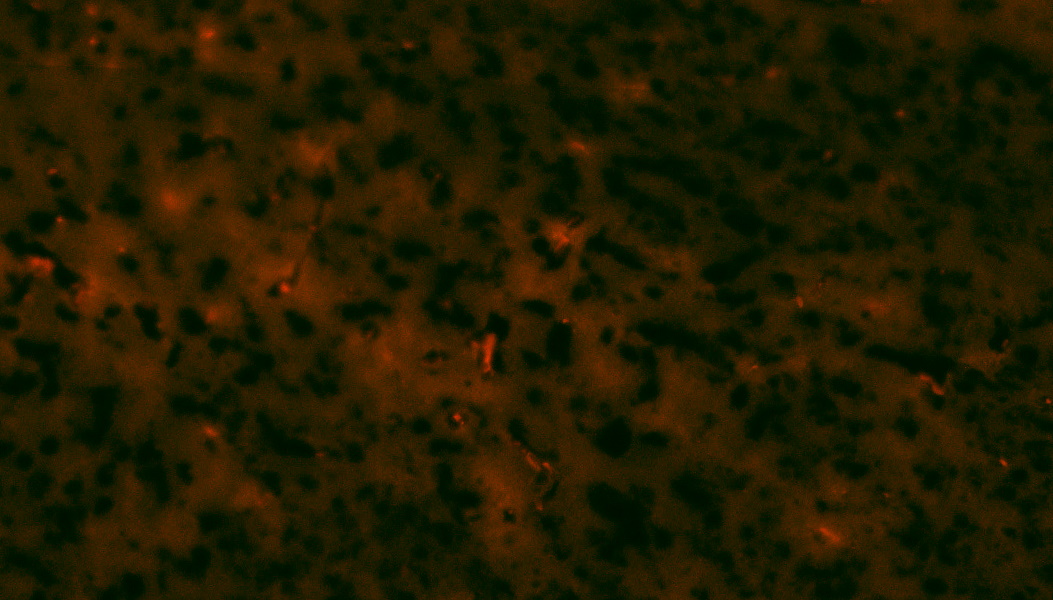

Supplement: Supplementary file 1 [file Data_Sheet_1.zip › Picture/Double Tracing/Left VPM-Merge.jpg]

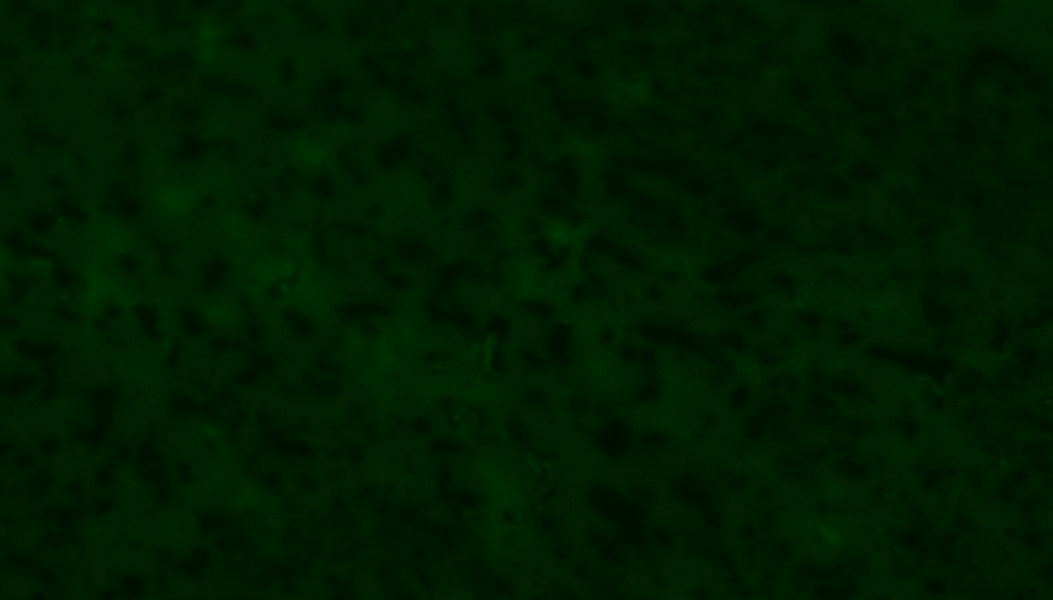

Supplement: Supplementary file 1 [file Data_Sheet_1.zip › Picture/Double Tracing/Left VPM-SpGreen.jpg]

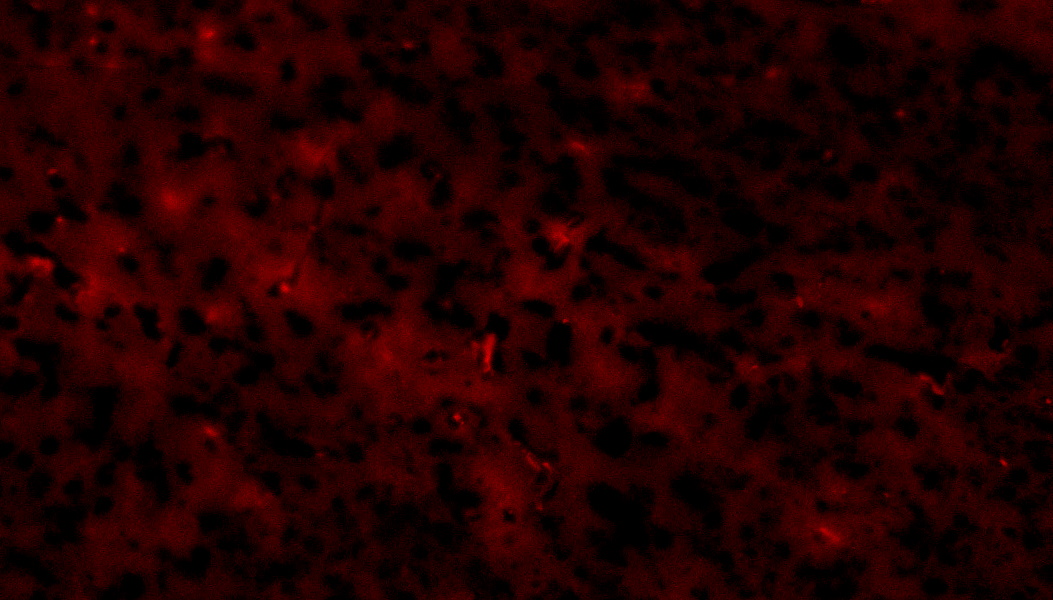

Supplement: Supplementary file 1 [file Data_Sheet_1.zip › Picture/Double Tracing/Left VPM-SpRed.jpg]

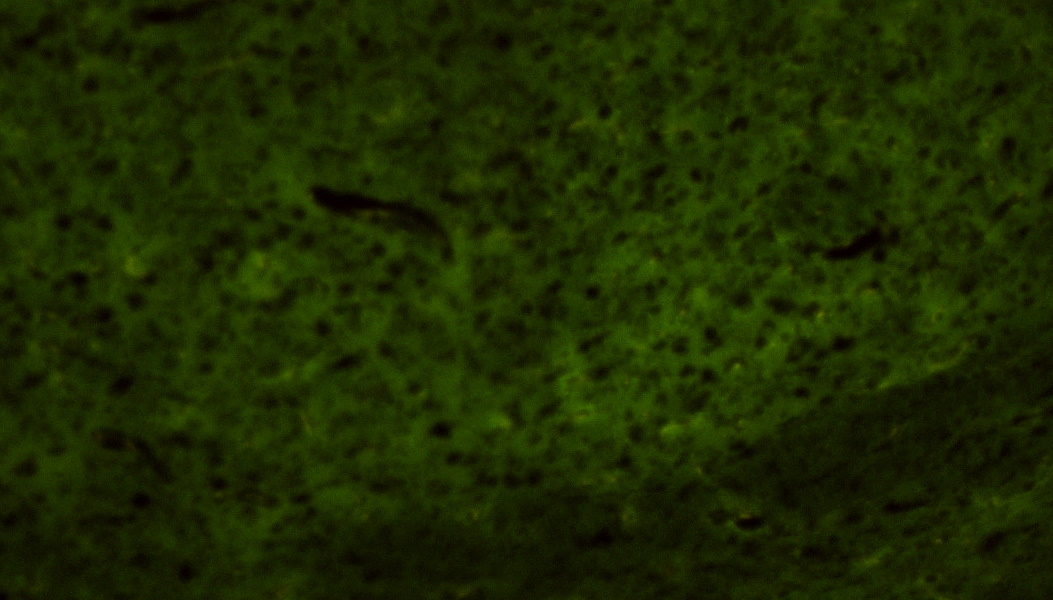

Supplement: Supplementary file 1 [file Data_Sheet_1.zip › Picture/Double Tracing/Right VPM-Merge.jpg]

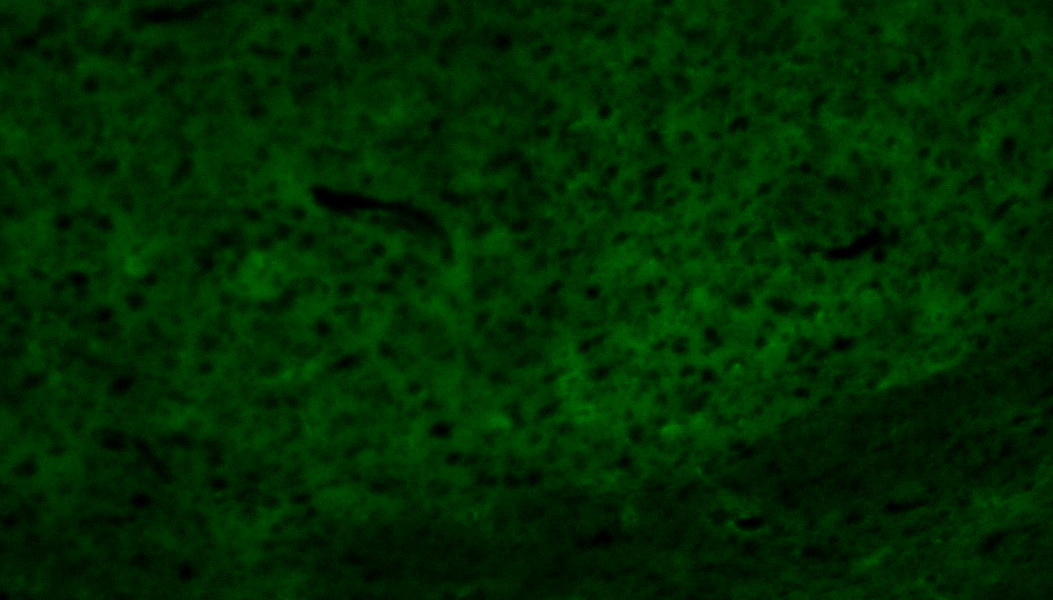

Supplement: Supplementary file 1 [file Data_Sheet_1.zip › Picture/Double Tracing/Right VPM-SpGreen.jpg]

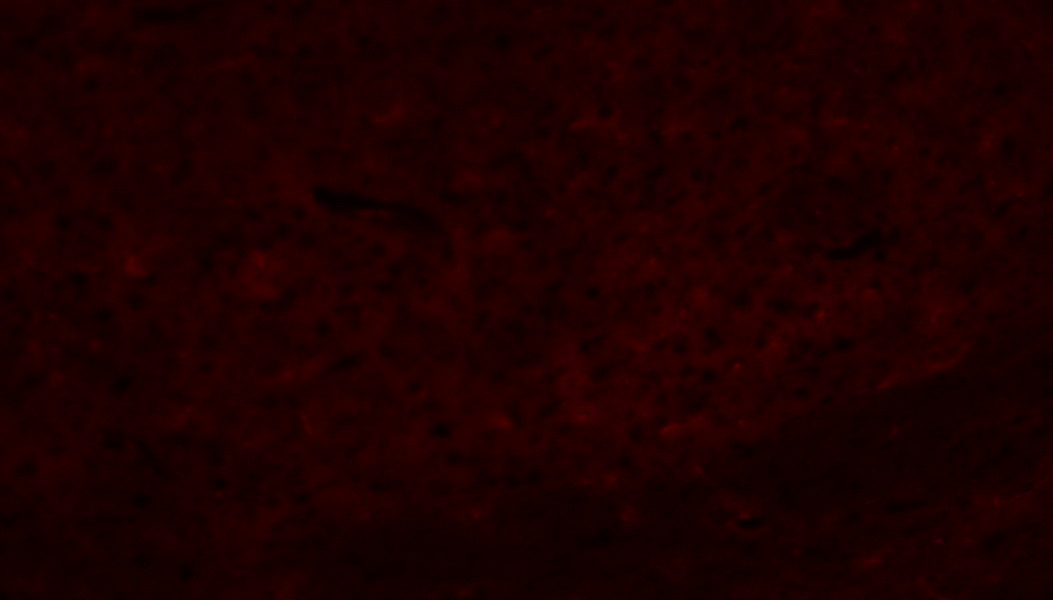

Supplement: Supplementary file 1 [file Data_Sheet_1.zip › Picture/Double Tracing/Right VPM-SpRed.jpg]

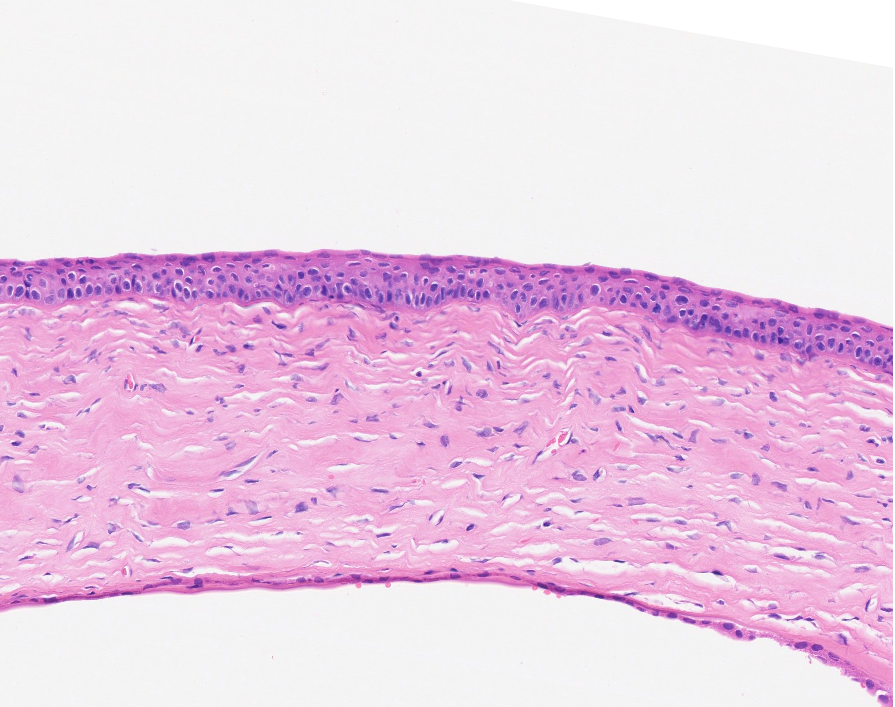

Supplement: Supplementary file 1 [file Data_Sheet_1.zip › Picture/HE-cornea/Acu-10.00X.tif]

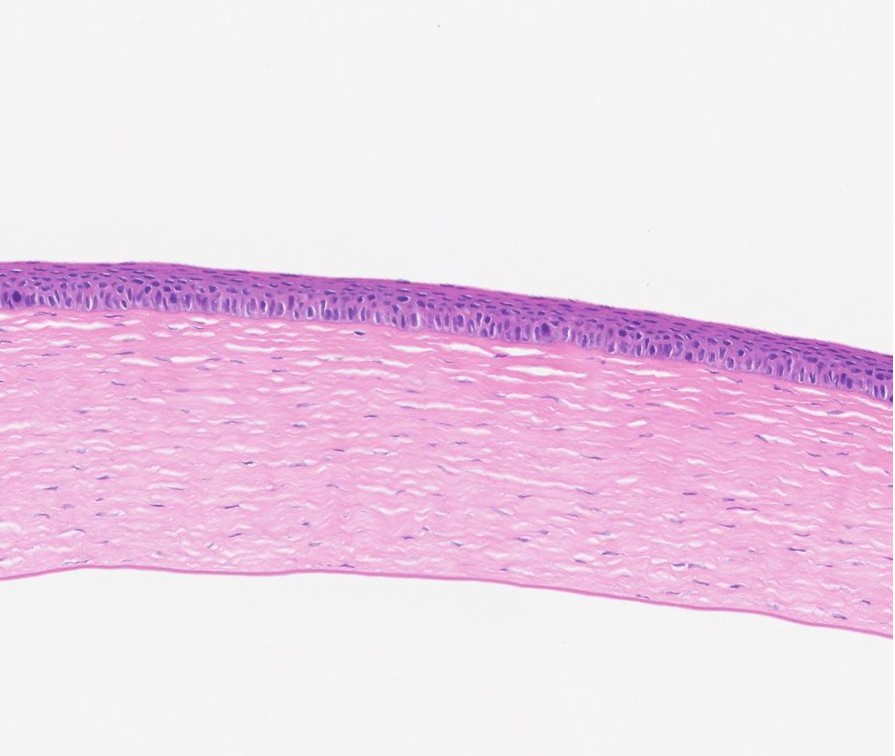

Supplement: Supplementary file 1 [file Data_Sheet_1.zip › Picture/HE-cornea/Con-10.00X.jpg]

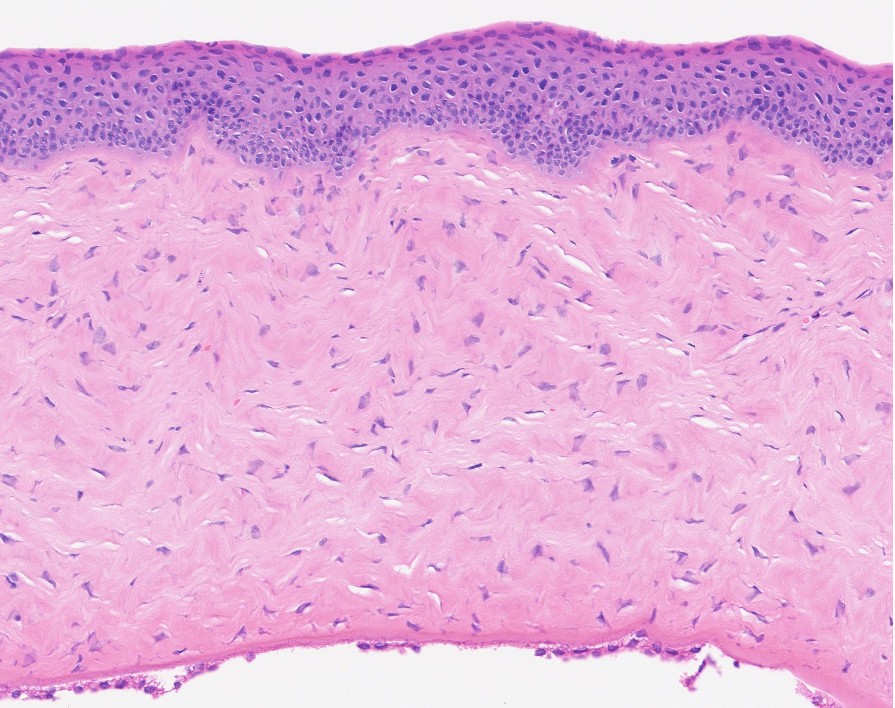

Supplement: Supplementary file 1 [file Data_Sheet_1.zip › Picture/HE-cornea/Mod-10.00X.jpg]

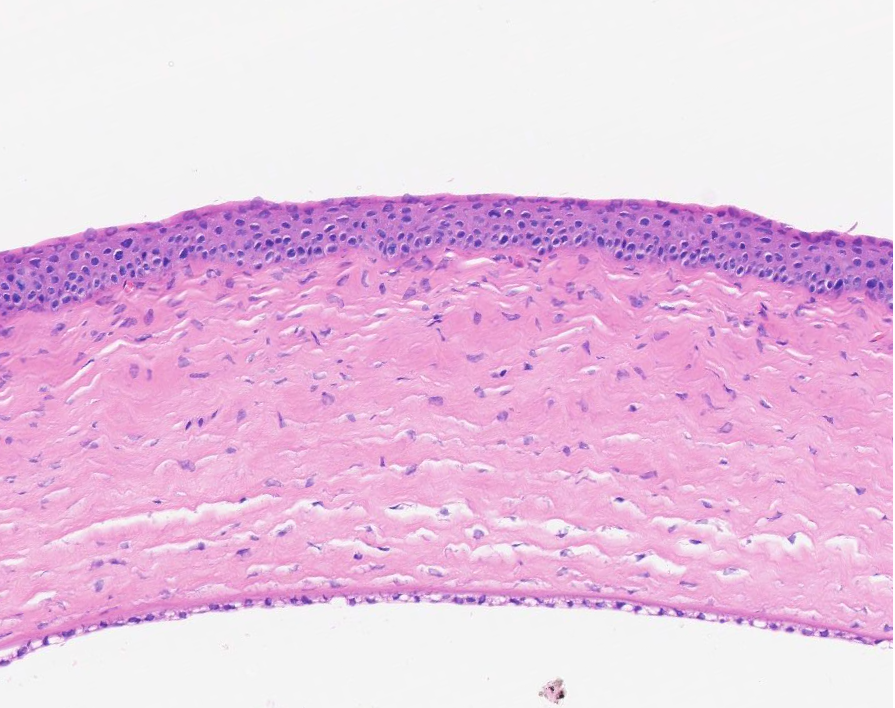

Supplement: Supplementary file 1 [file Data_Sheet_1.zip › Picture/HE-cornea/NA-10.00X.tif]

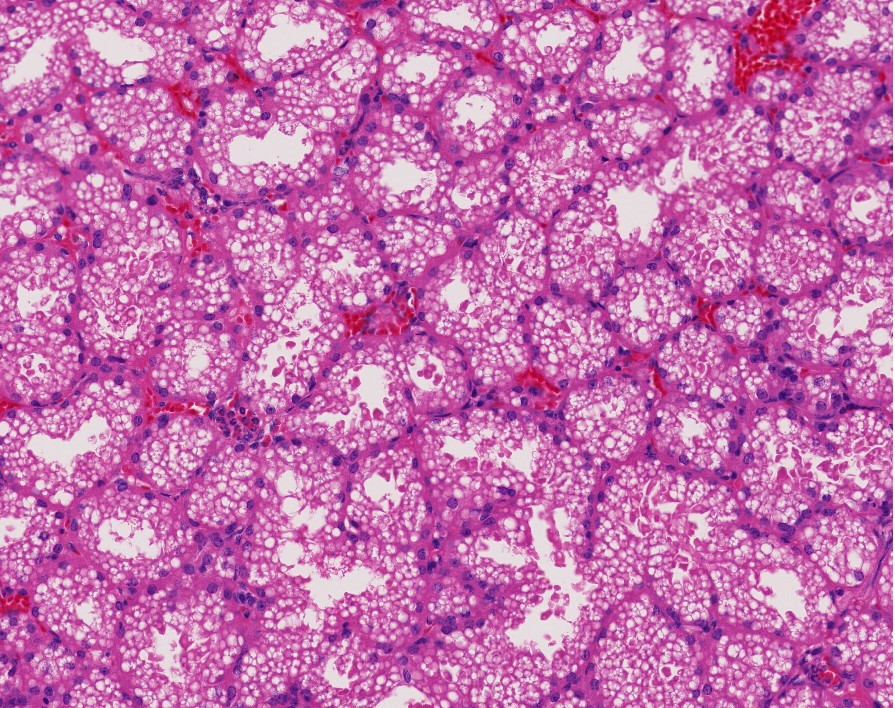

Supplement: Supplementary file 1 [file Data_Sheet_1.zip › Picture/HE-lacrymal gland/Acu-10.00X.jpg]

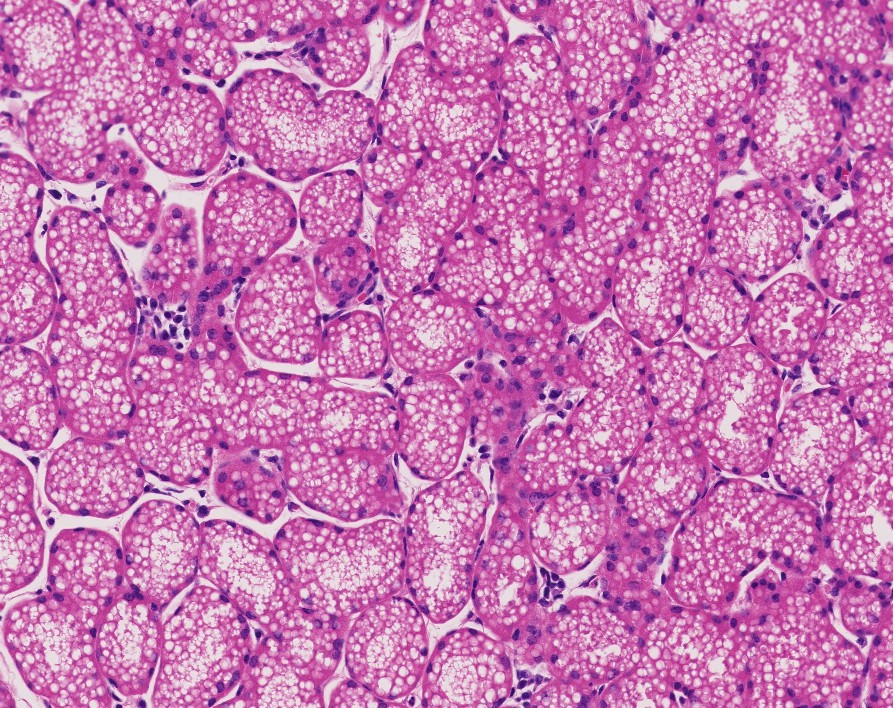

Supplement: Supplementary file 1 [file Data_Sheet_1.zip › Picture/HE-lacrymal gland/Con-10.00X.jpg]

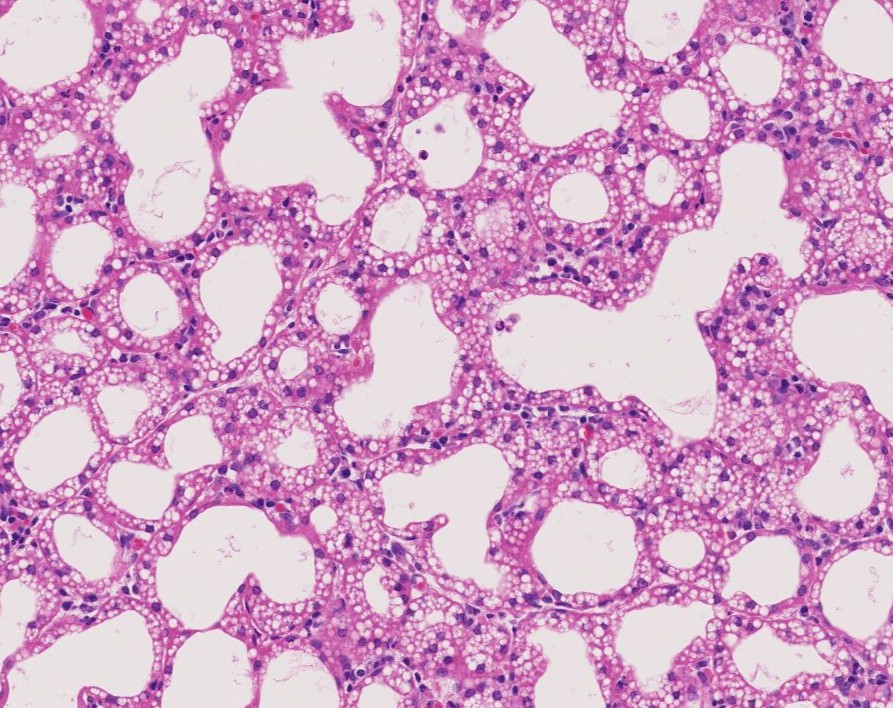

Supplement: Supplementary file 1 [file Data_Sheet_1.zip › Picture/HE-lacrymal gland/Mod-10.00X.jpg]

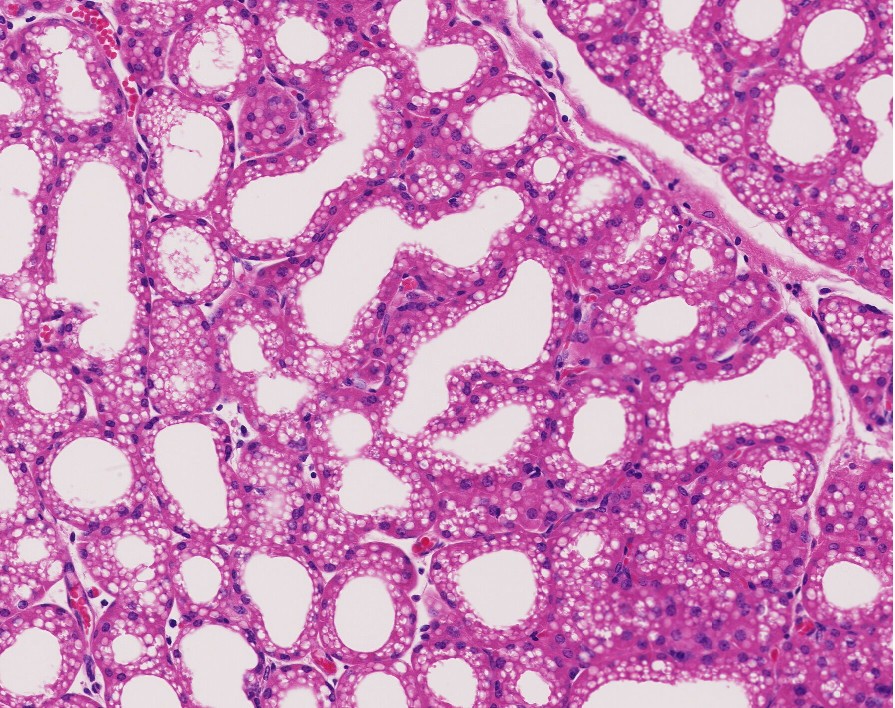

Supplement: Supplementary file 1 [file Data_Sheet_1.zip › Picture/HE-lacrymal gland/NA-10.00X.jpg]

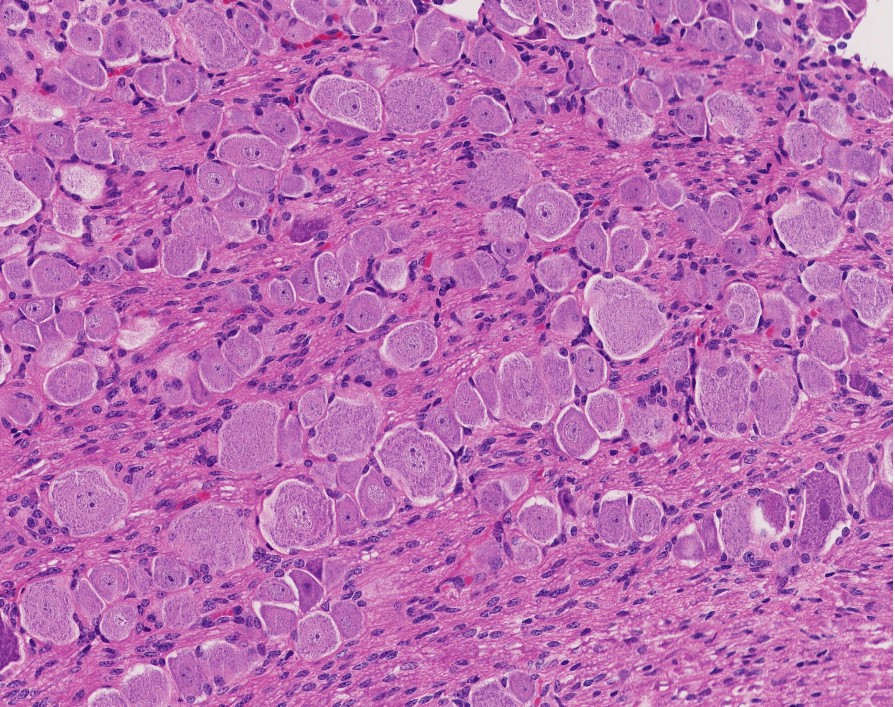

Supplement: Supplementary file 1 [file Data_Sheet_1.zip › Picture/HE-trigeminal nerve/Acu-10.00X.jpg]

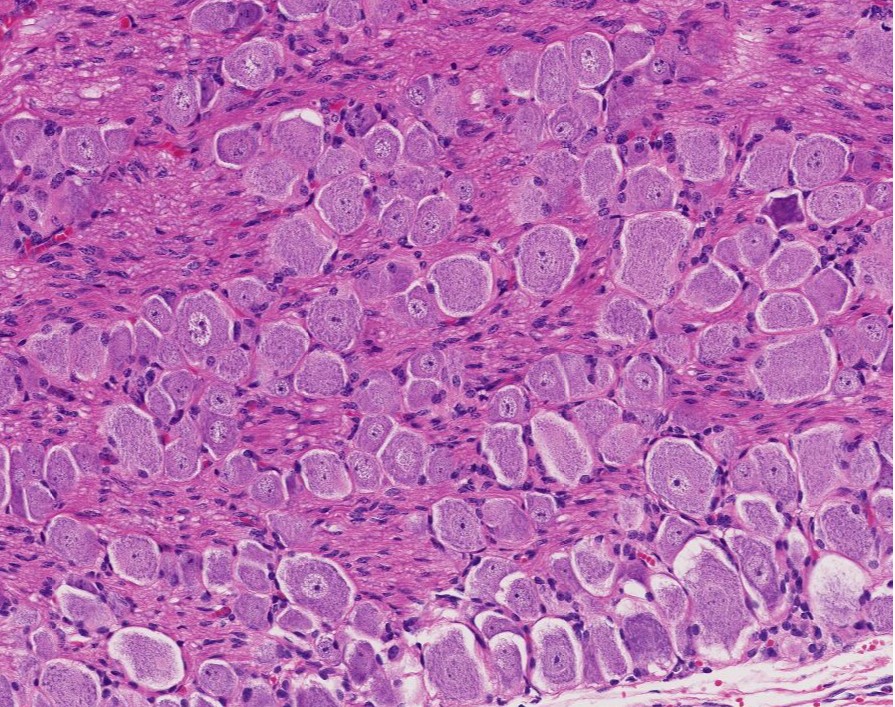

Supplement: Supplementary file 1 [file Data_Sheet_1.zip › Picture/HE-trigeminal nerve/Con-10.00X.jpg]

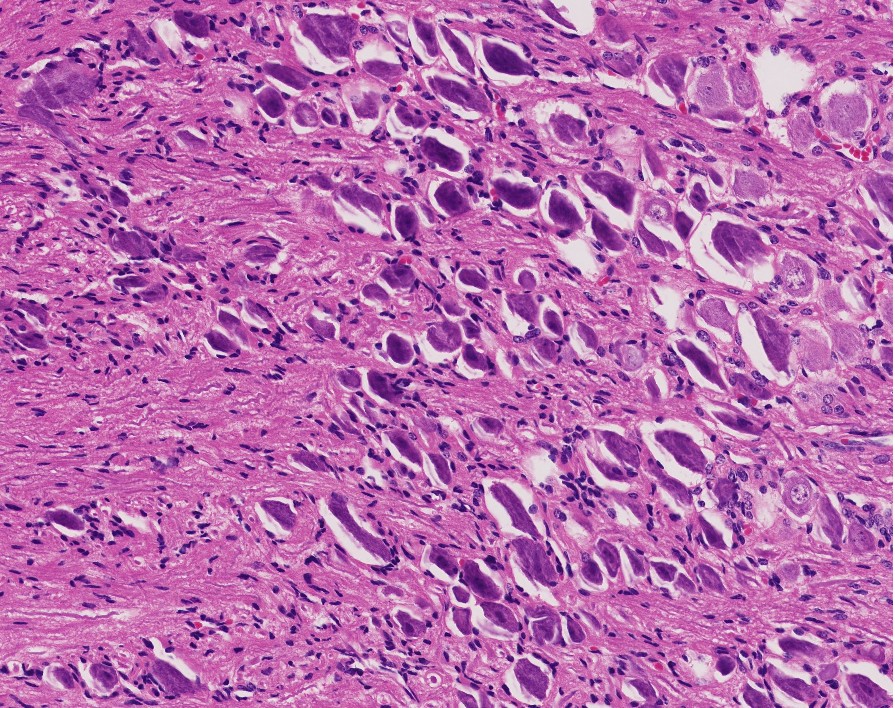

Supplement: Supplementary file 1 [file Data_Sheet_1.zip › Picture/HE-trigeminal nerve/Mod-10.00X.jpg]

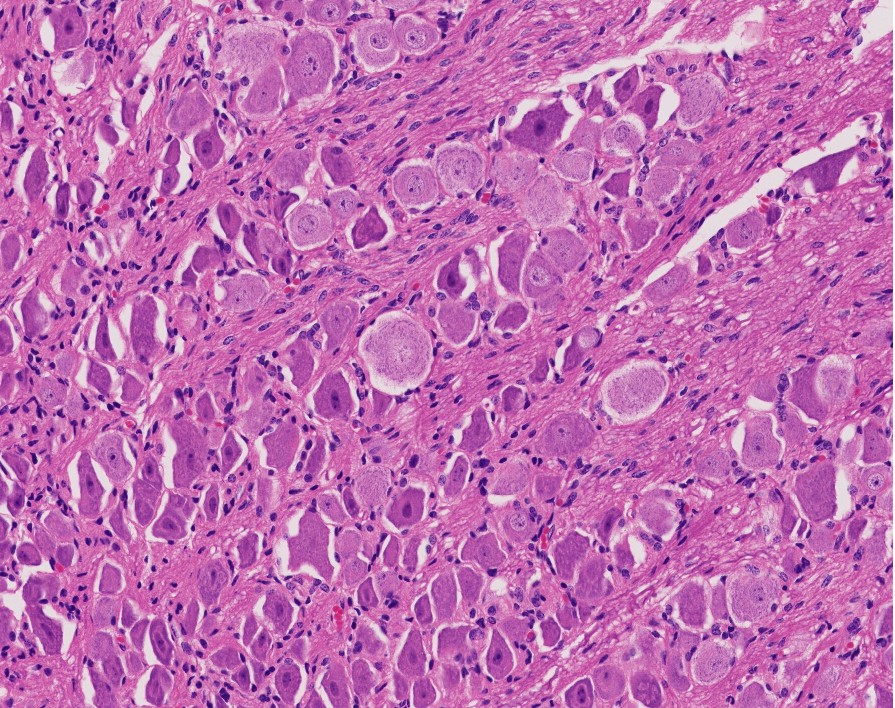

Supplement: Supplementary file 1 [file Data_Sheet_1.zip › Picture/HE-trigeminal nerve/NA-10.00X.jpg]

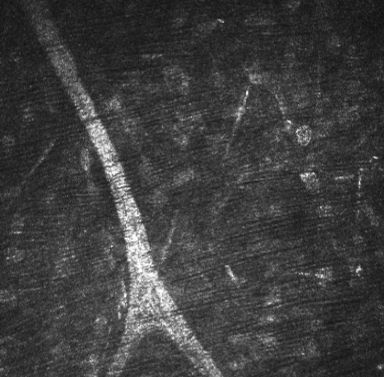

Supplement: Supplementary file 1 [file Data_Sheet_1.zip › Picture/IVCM/Acu.tif]

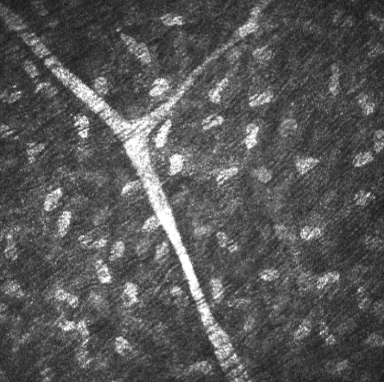

Supplement: Supplementary file 1 [file Data_Sheet_1.zip › Picture/IVCM/Con.tif]

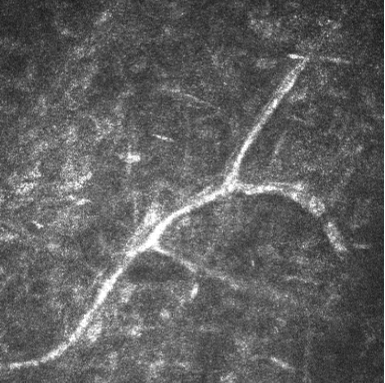

Supplement: Supplementary file 1 [file Data_Sheet_1.zip › Picture/IVCM/Mod.tif]

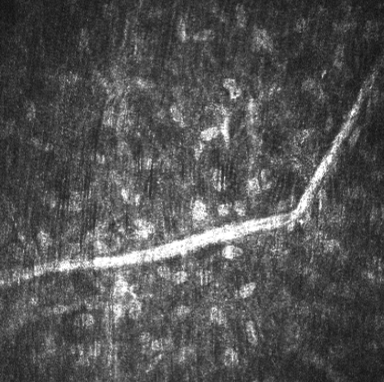

Supplement: Supplementary file 1 [file Data_Sheet_1.zip › Picture/IVCM/NA.tif]

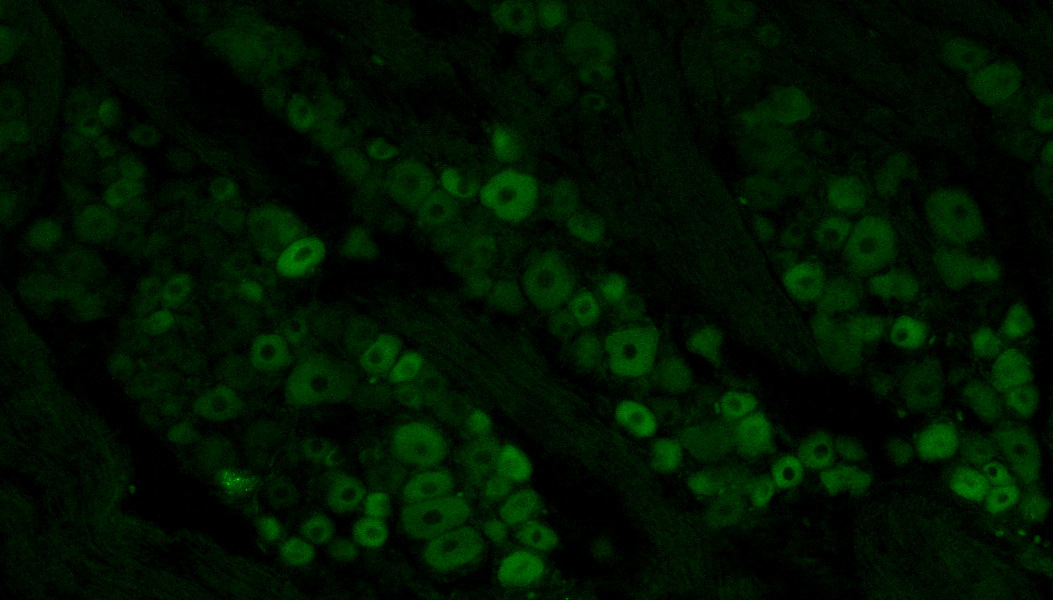

Supplement: Supplementary file 1 [file Data_Sheet_1.zip › Picture/Single Tracing-trigeminal nerve/BL1-20.0X.jpg]

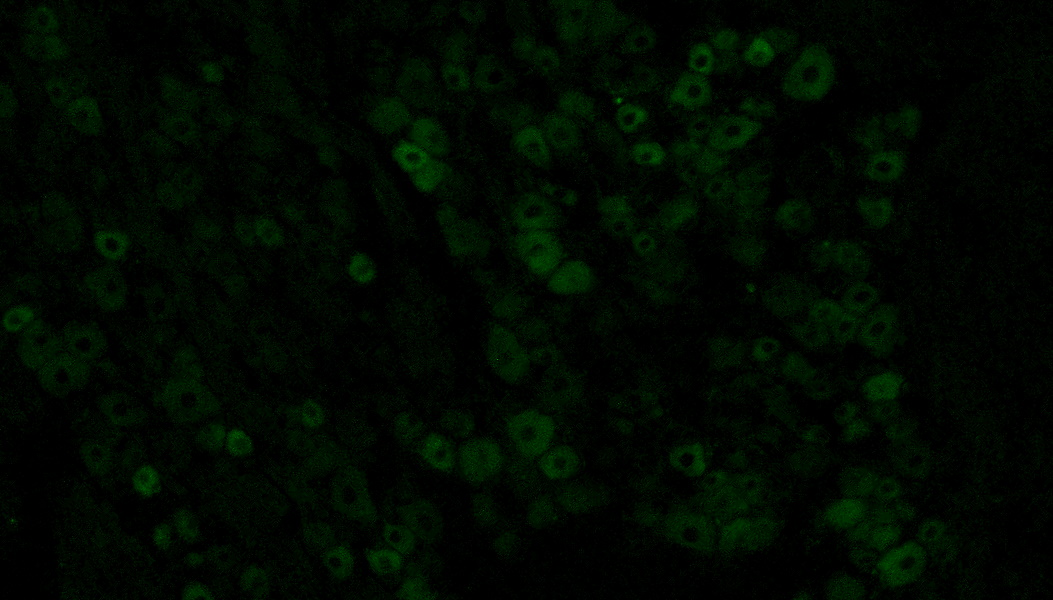

Supplement: Supplementary file 1 [file Data_Sheet_1.zip › Picture/Single Tracing-trigeminal nerve/BL2-20.0X.jpg]

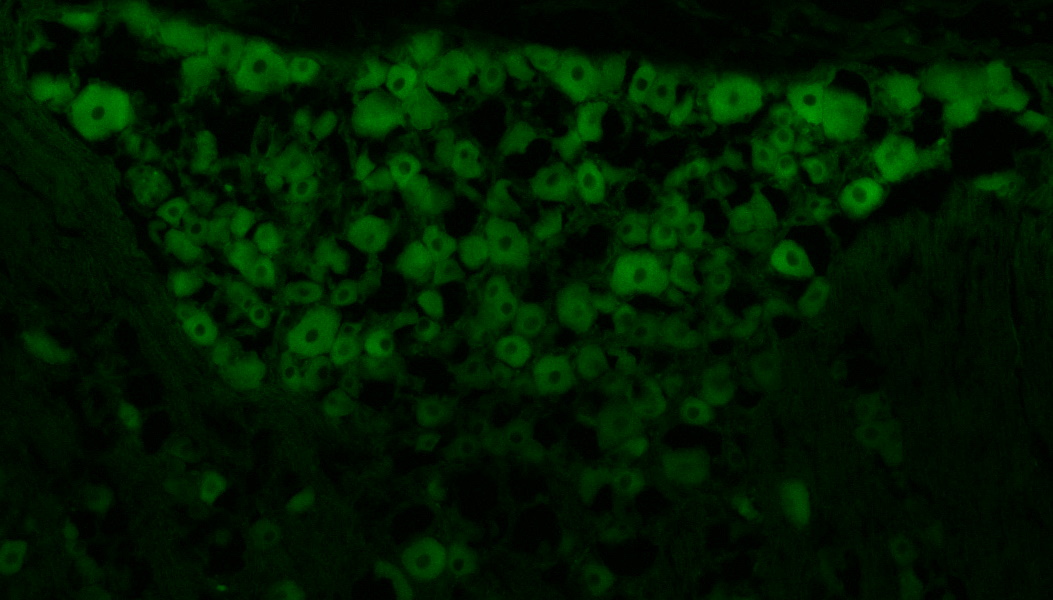

Supplement: Supplementary file 1 [file Data_Sheet_1.zip › Picture/Single Tracing-trigeminal nerve/EX-HN5-20.0X.jpg]

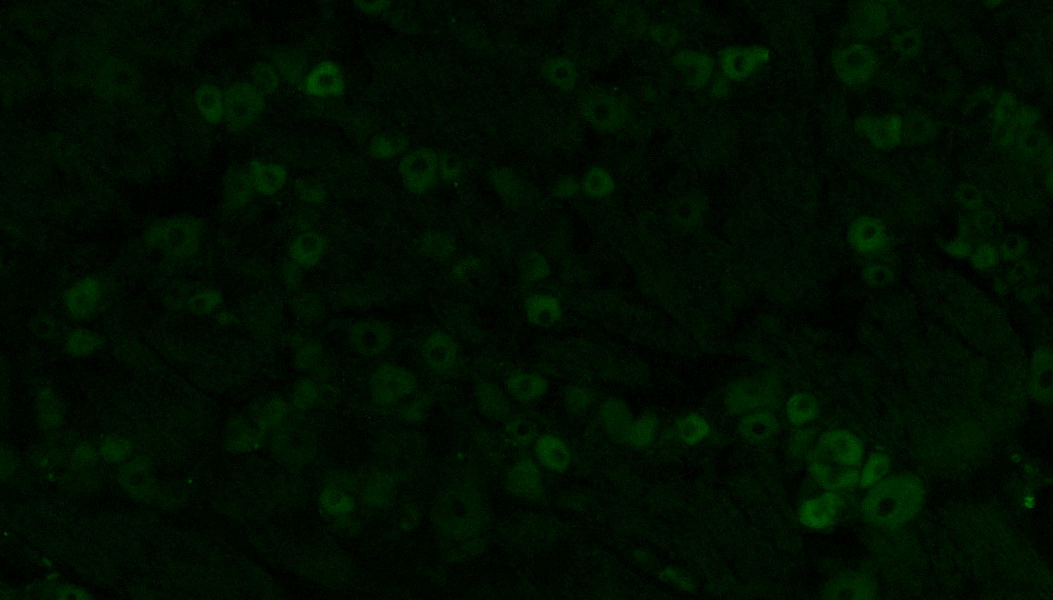

Supplement: Supplementary file 1 [file Data_Sheet_1.zip › Picture/Single Tracing-trigeminal nerve/GB1-20.0X.jpg]

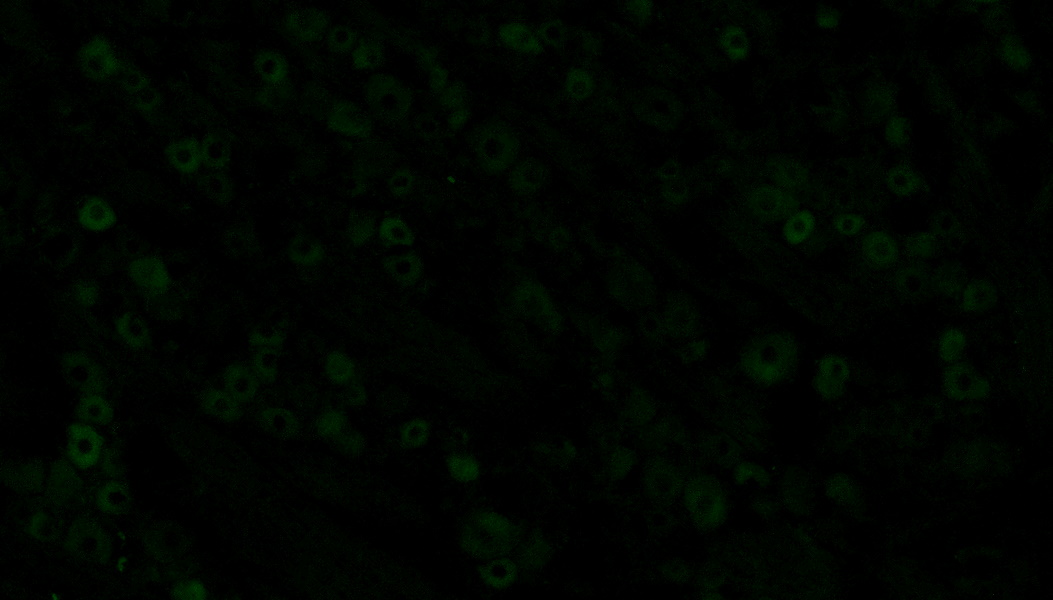

Supplement: Supplementary file 1 [file Data_Sheet_1.zip › Picture/Single Tracing-trigeminal nerve/N1-20.0X.jpg]

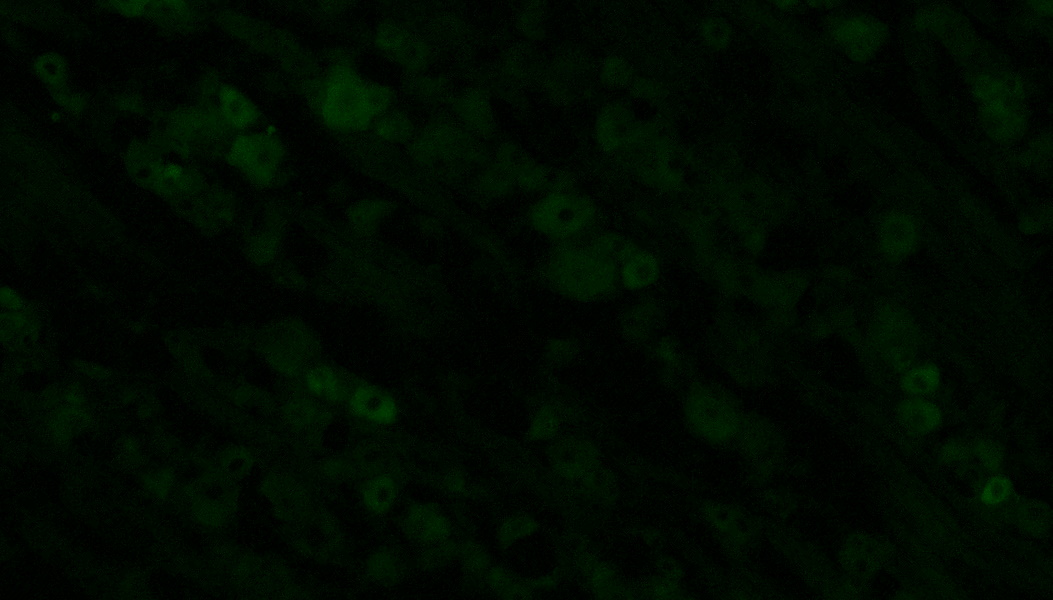

Supplement: Supplementary file 1 [file Data_Sheet_1.zip › Picture/Single Tracing-trigeminal nerve/N2-20.0X.jpg]

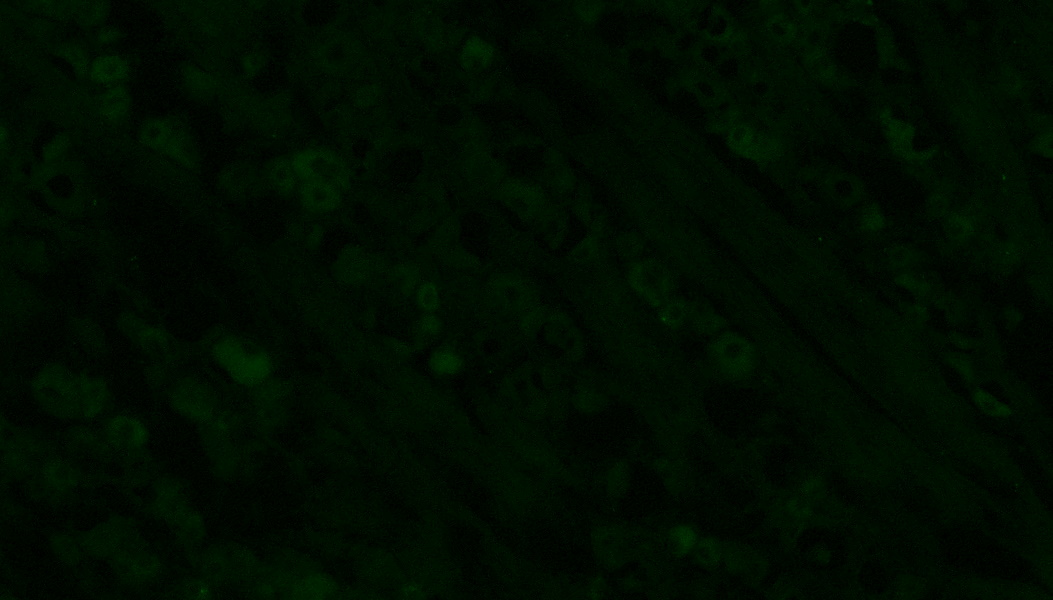

Supplement: Supplementary file 1 [file Data_Sheet_1.zip › Picture/Single Tracing-trigeminal nerve/N3-20.0X.jpg]

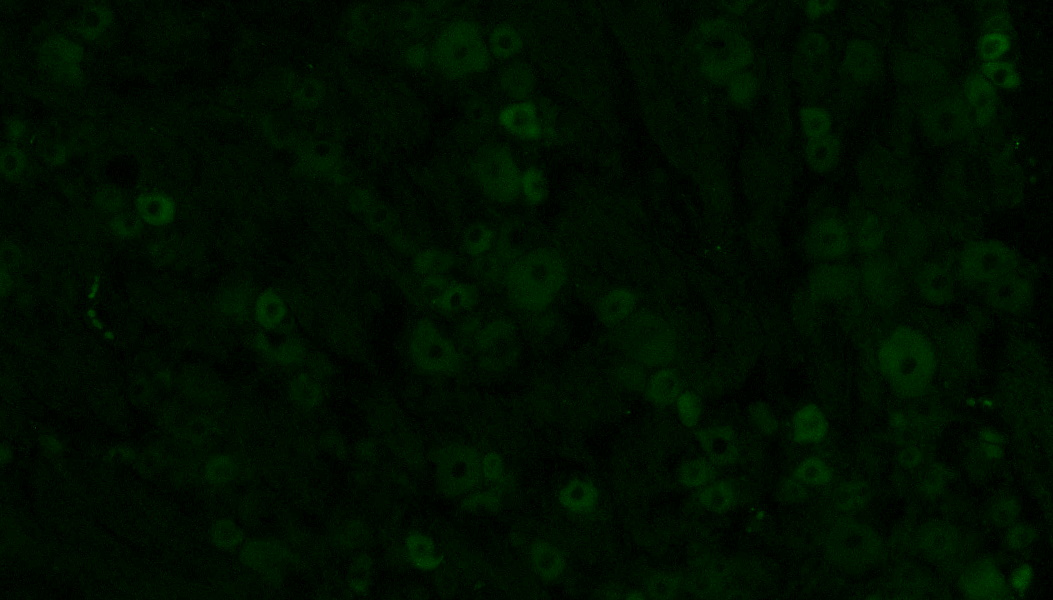

Supplement: Supplementary file 1 [file Data_Sheet_1.zip › Picture/Single Tracing-trigeminal nerve/N4-20.0X.jpg]

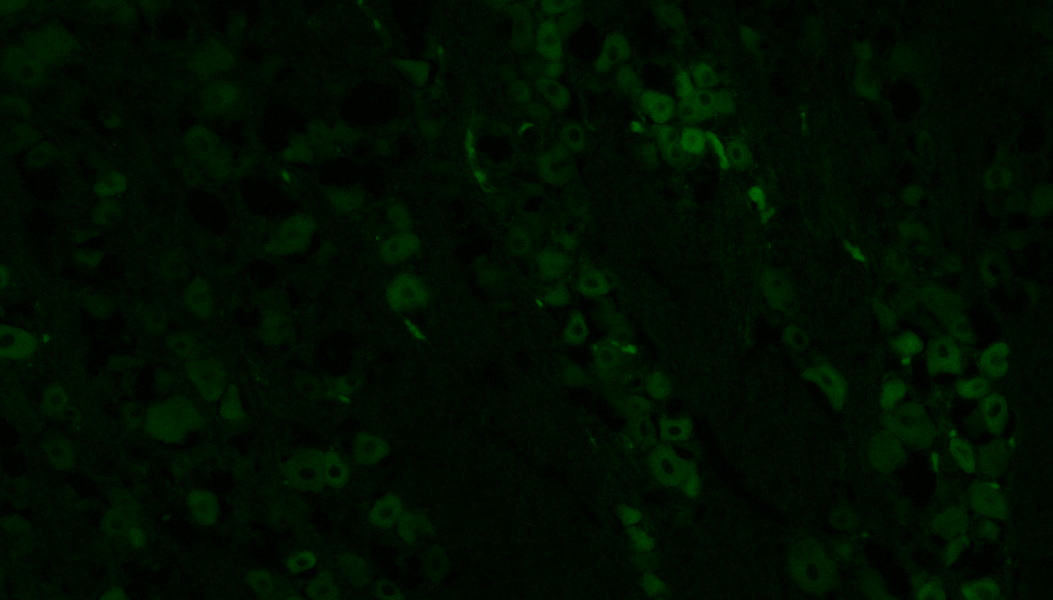

Supplement: Supplementary file 1 [file Data_Sheet_1.zip › Picture/Single Tracing-trigeminal nerve/N5-20.0X.jpg]

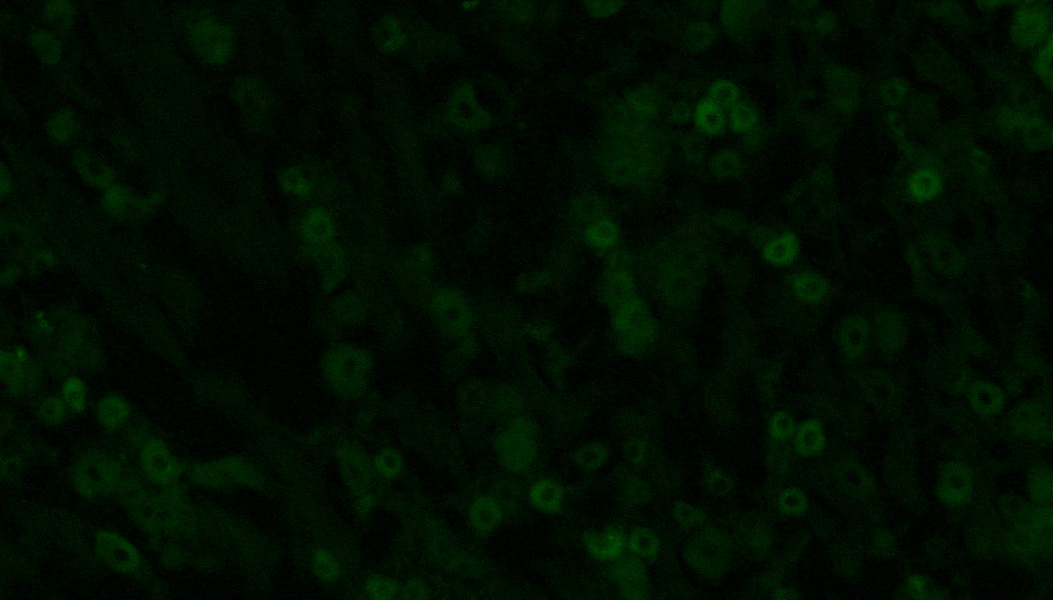

Supplement: Supplementary file 1 [file Data_Sheet_1.zip › Picture/Single Tracing-trigeminal nerve/SJ23-20.0X.jpg]

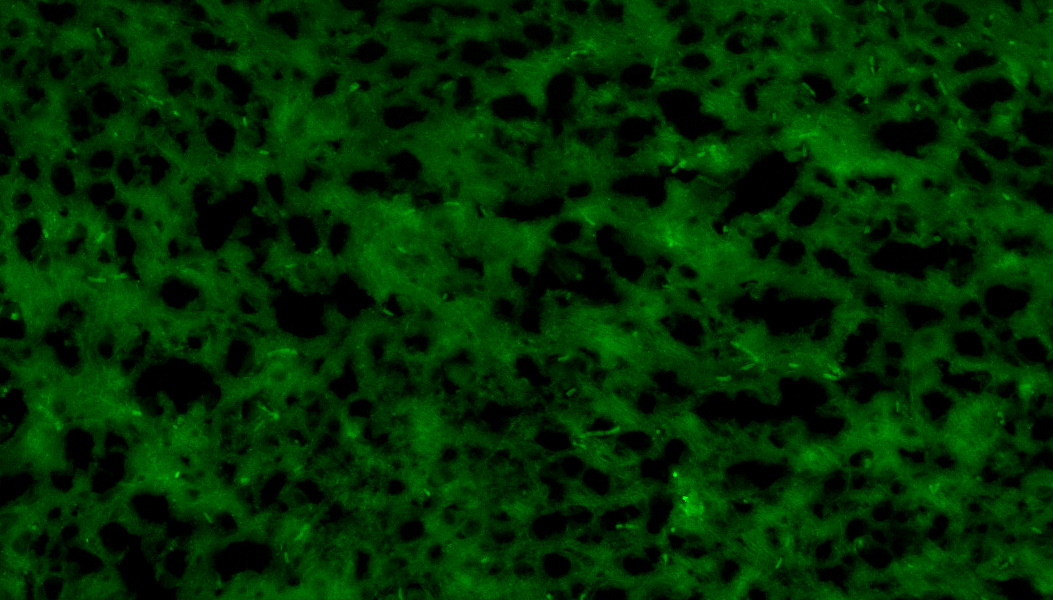

Supplement: Supplementary file 1 [file Data_Sheet_1.zip › Picture/Single Tracing-VPM/BL1-20.00X.jpg]

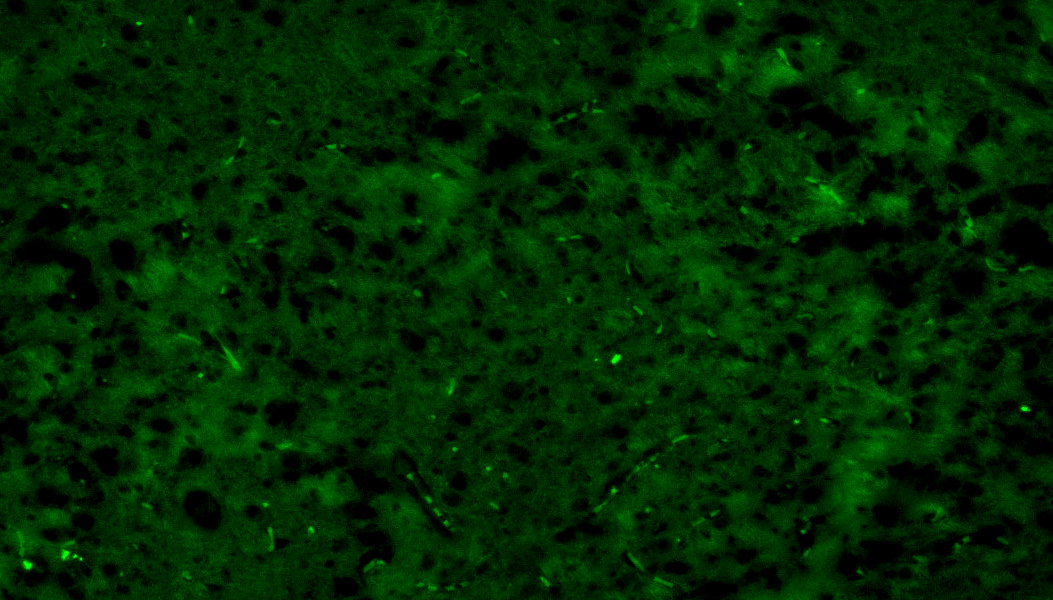

Supplement: Supplementary file 1 [file Data_Sheet_1.zip › Picture/Single Tracing-VPM/BL2-20.00X.jpg]

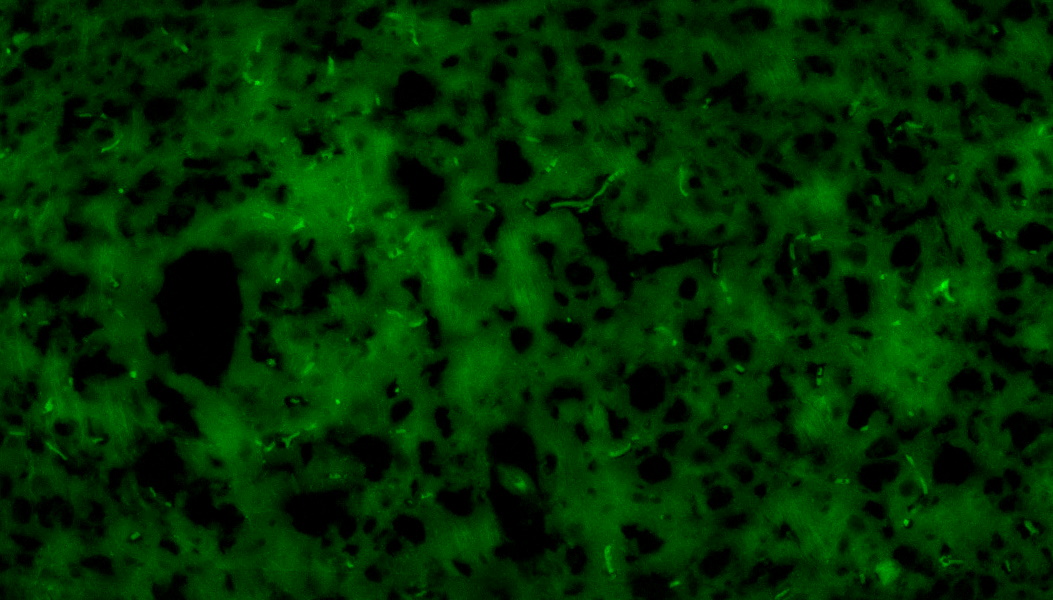

Supplement: Supplementary file 1 [file Data_Sheet_1.zip › Picture/Single Tracing-VPM/EX-HN5-20.00X.jpg]

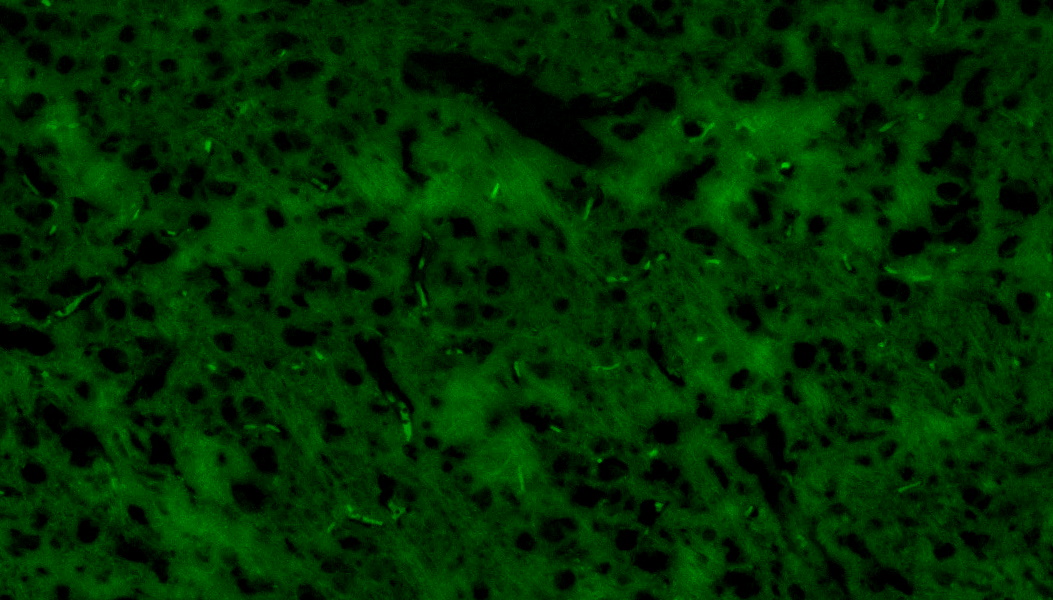

Supplement: Supplementary file 1 [file Data_Sheet_1.zip › Picture/Single Tracing-VPM/GB1-20.00X.jpg]

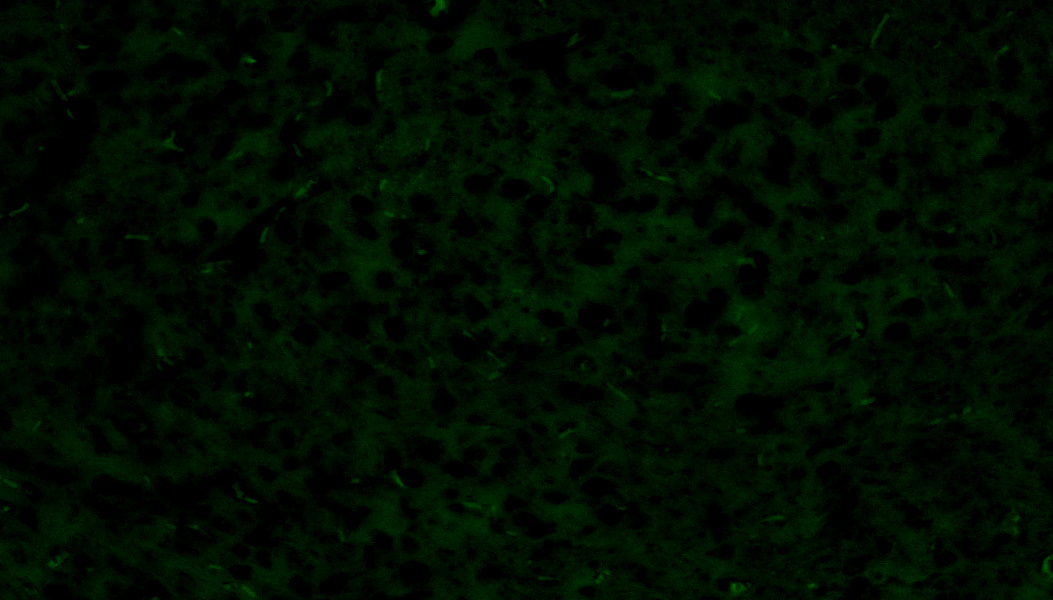

Supplement: Supplementary file 1 [file Data_Sheet_1.zip › Picture/Single Tracing-VPM/N1-20.00X.jpg]

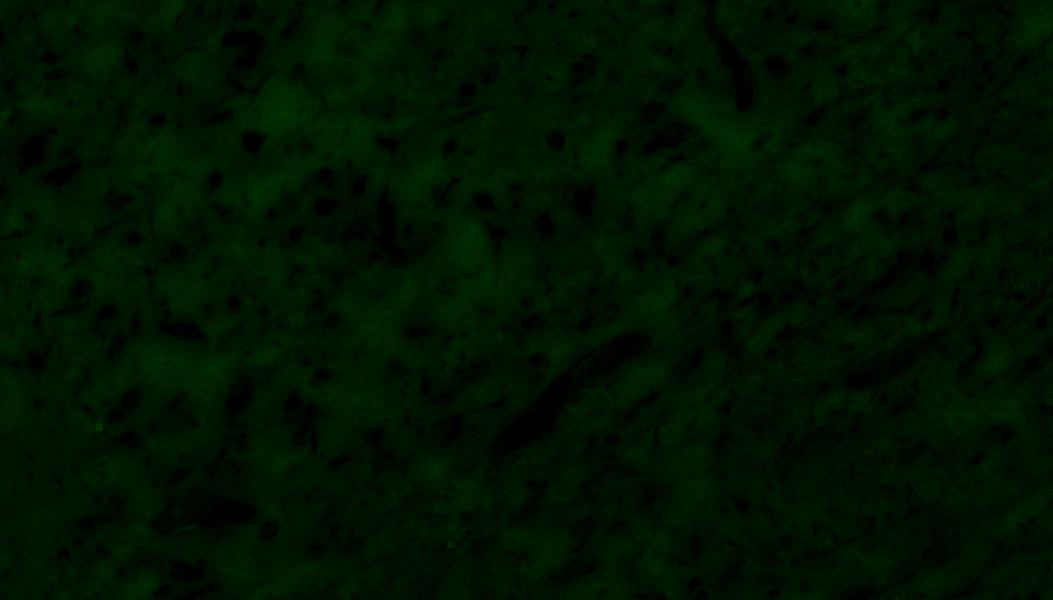

Supplement: Supplementary file 1 [file Data_Sheet_1.zip › Picture/Single Tracing-VPM/N2-20.00X.jpg]

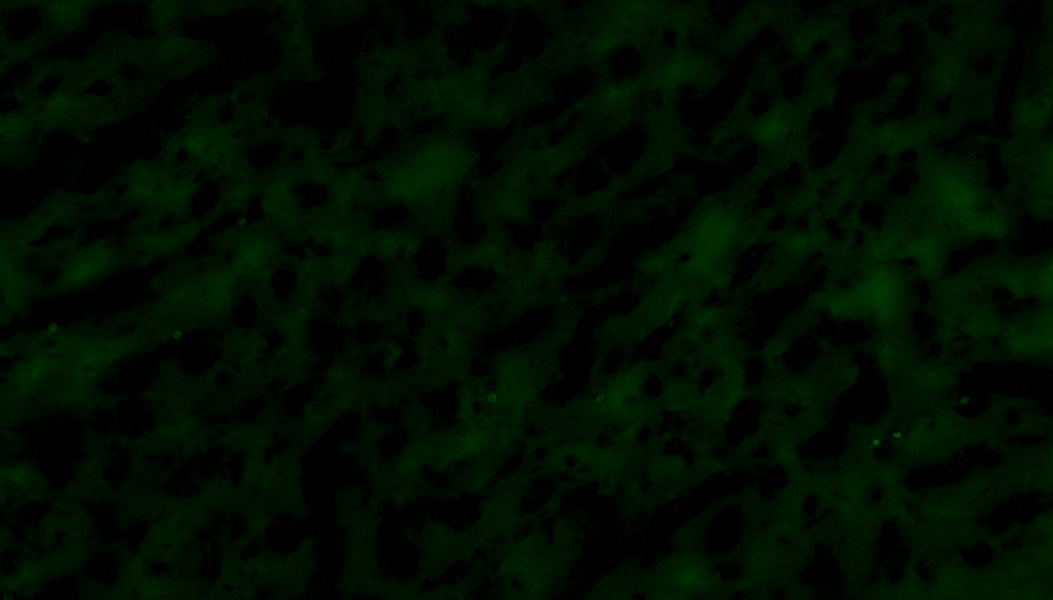

Supplement: Supplementary file 1 [file Data_Sheet_1.zip › Picture/Single Tracing-VPM/N3-20.00X.jpg]

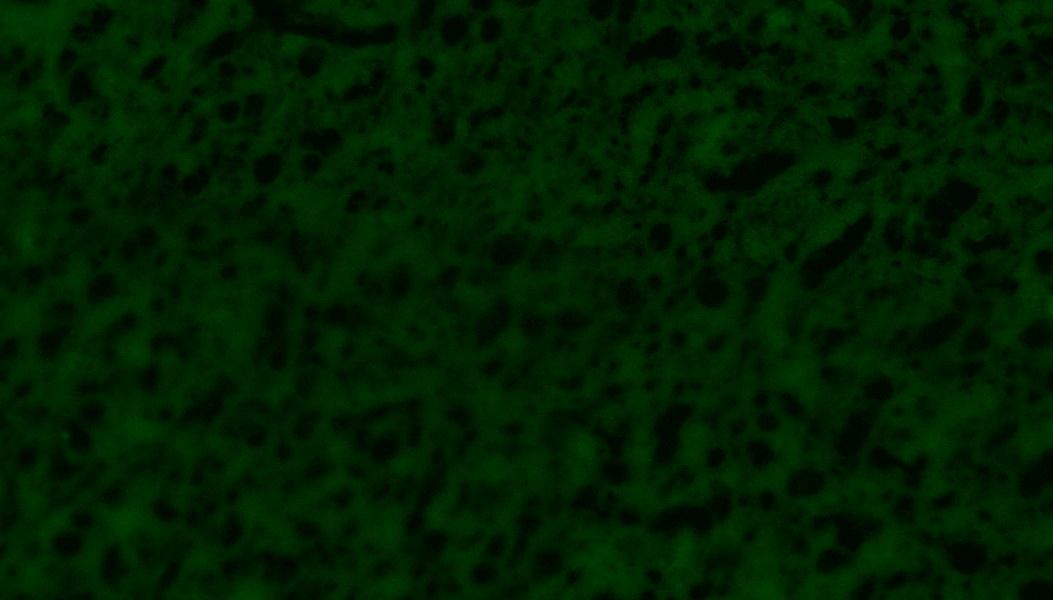

Supplement: Supplementary file 1 [file Data_Sheet_1.zip › Picture/Single Tracing-VPM/N4-20.00X.jpg]

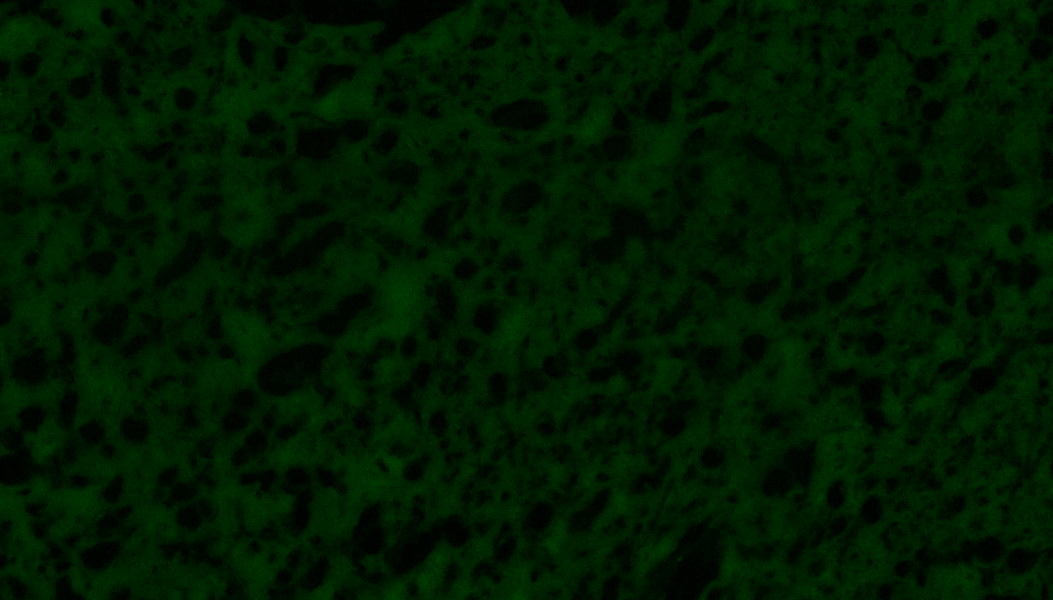

Supplement: Supplementary file 1 [file Data_Sheet_1.zip › Picture/Single Tracing-VPM/N5-20.00X.jpg]

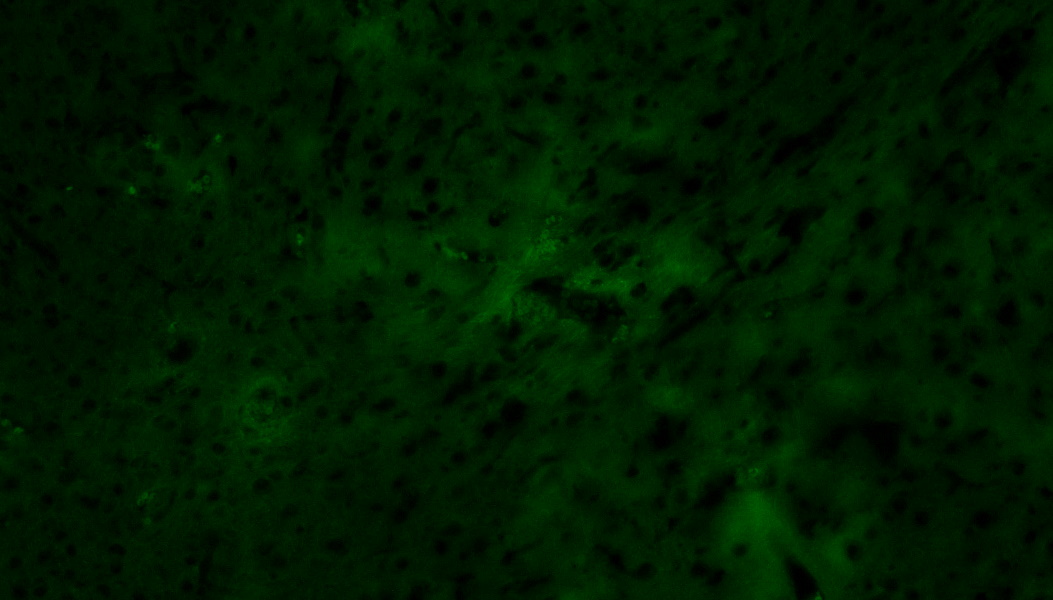

Supplement: Supplementary file 1 [file Data_Sheet_1.zip › Picture/Single Tracing-VPM/SJ23-20.00X.jpg]

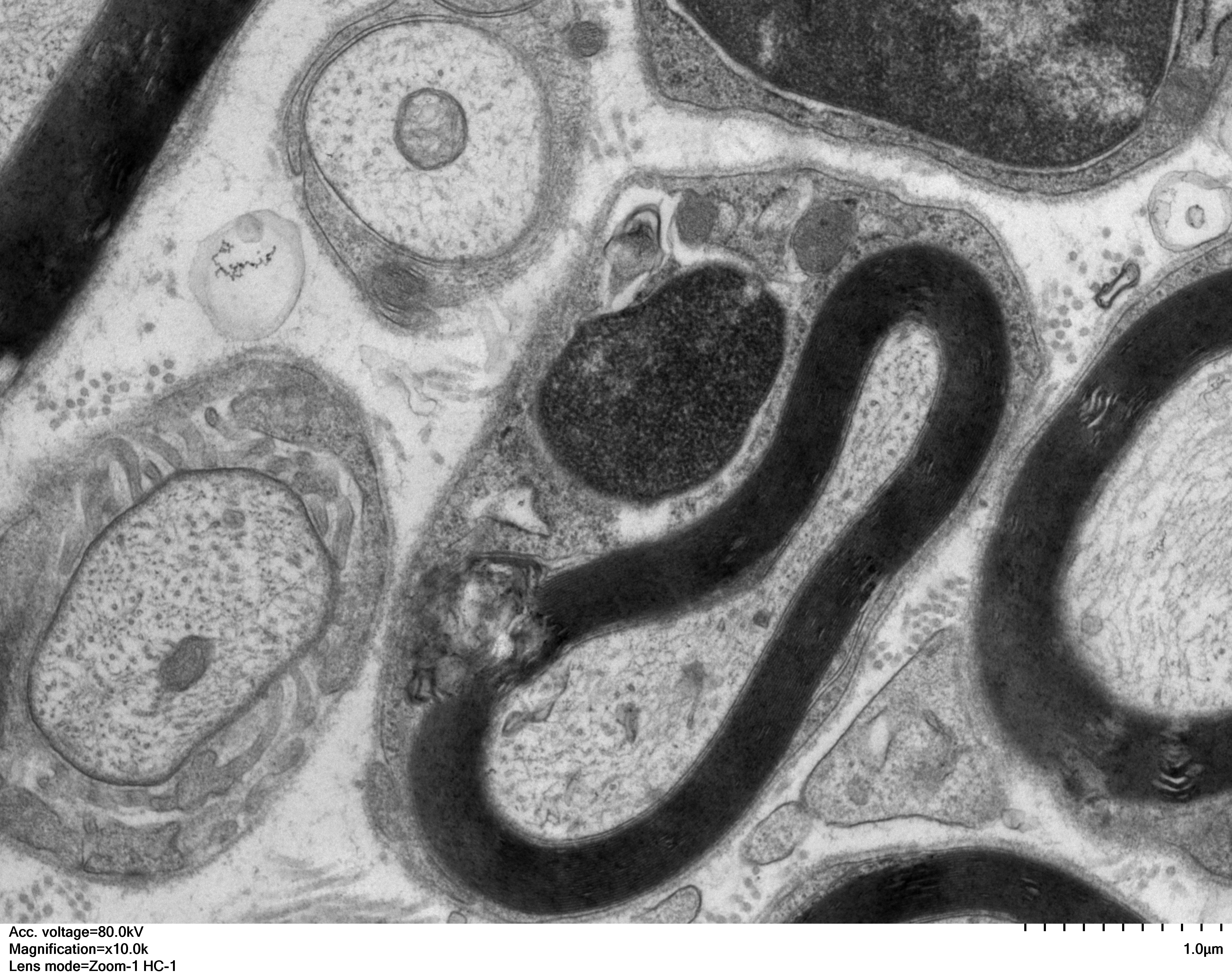

Supplement: Supplementary file 1 [file Data_Sheet_1.zip › Picture/TEM-trigeminal nerve/Acu-.jpg]

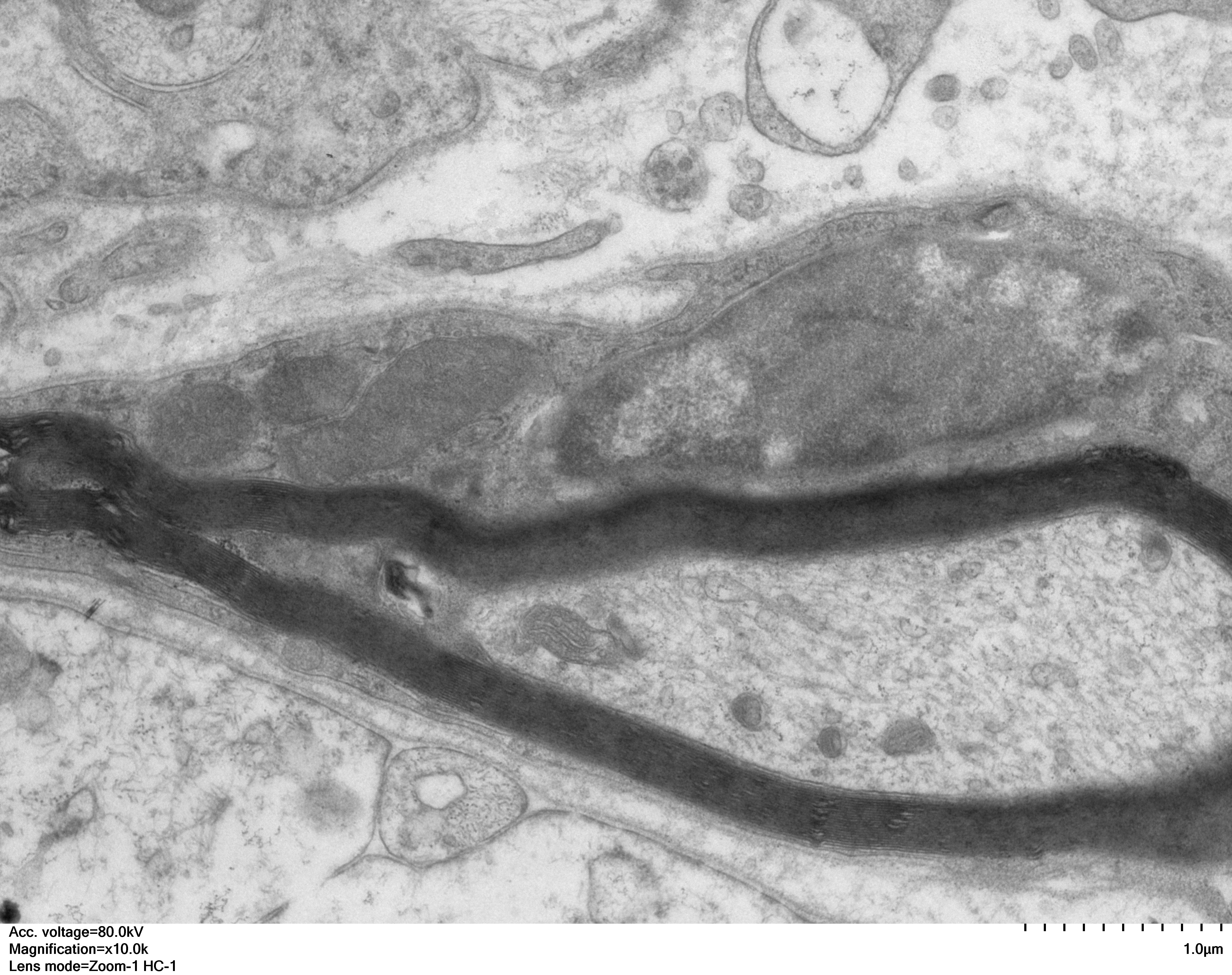

Supplement: Supplementary file 1 [file Data_Sheet_1.zip › Picture/TEM-trigeminal nerve/Con-.jpg]

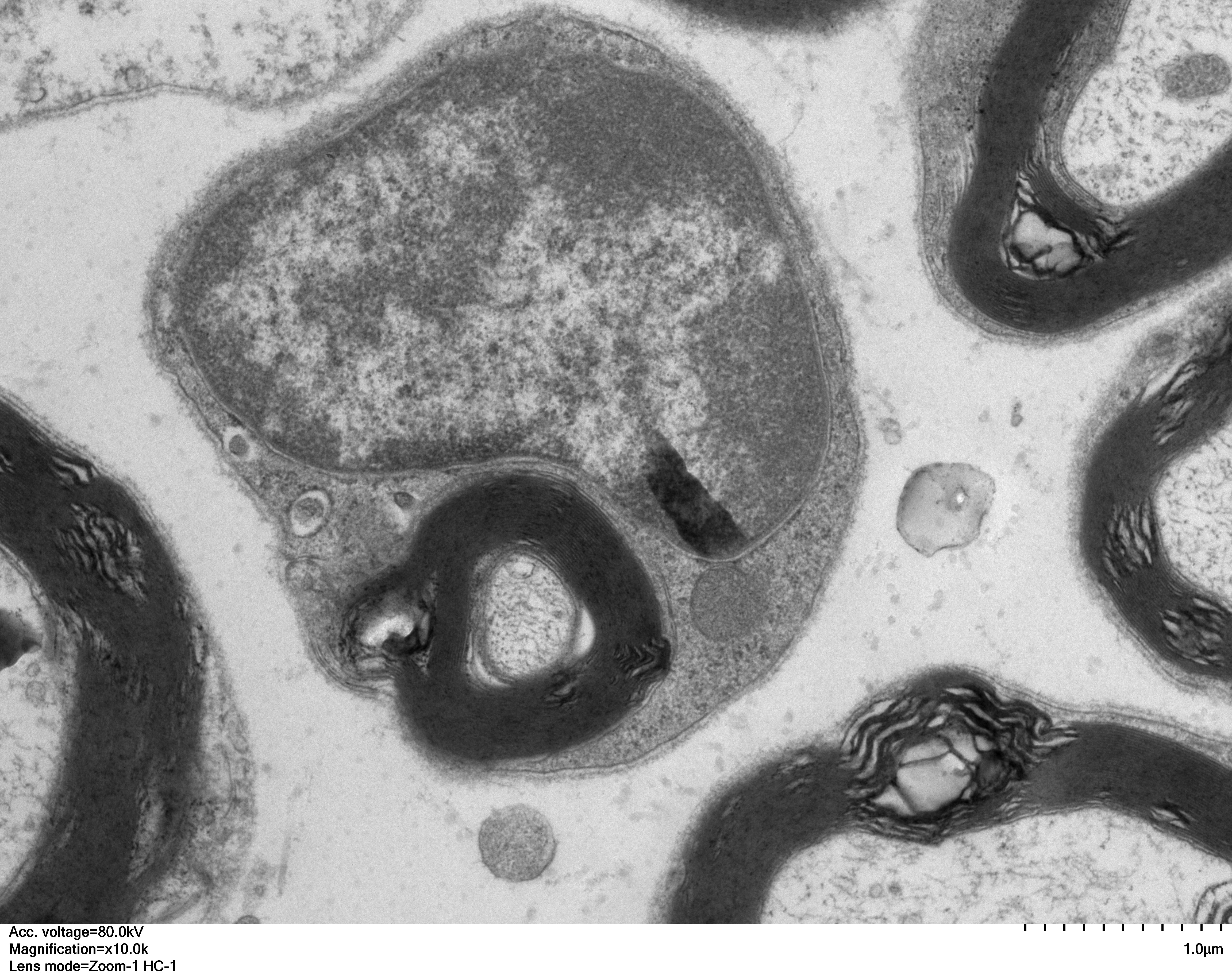

Supplement: Supplementary file 1 [file Data_Sheet_1.zip › Picture/TEM-trigeminal nerve/Mod-.jpg]

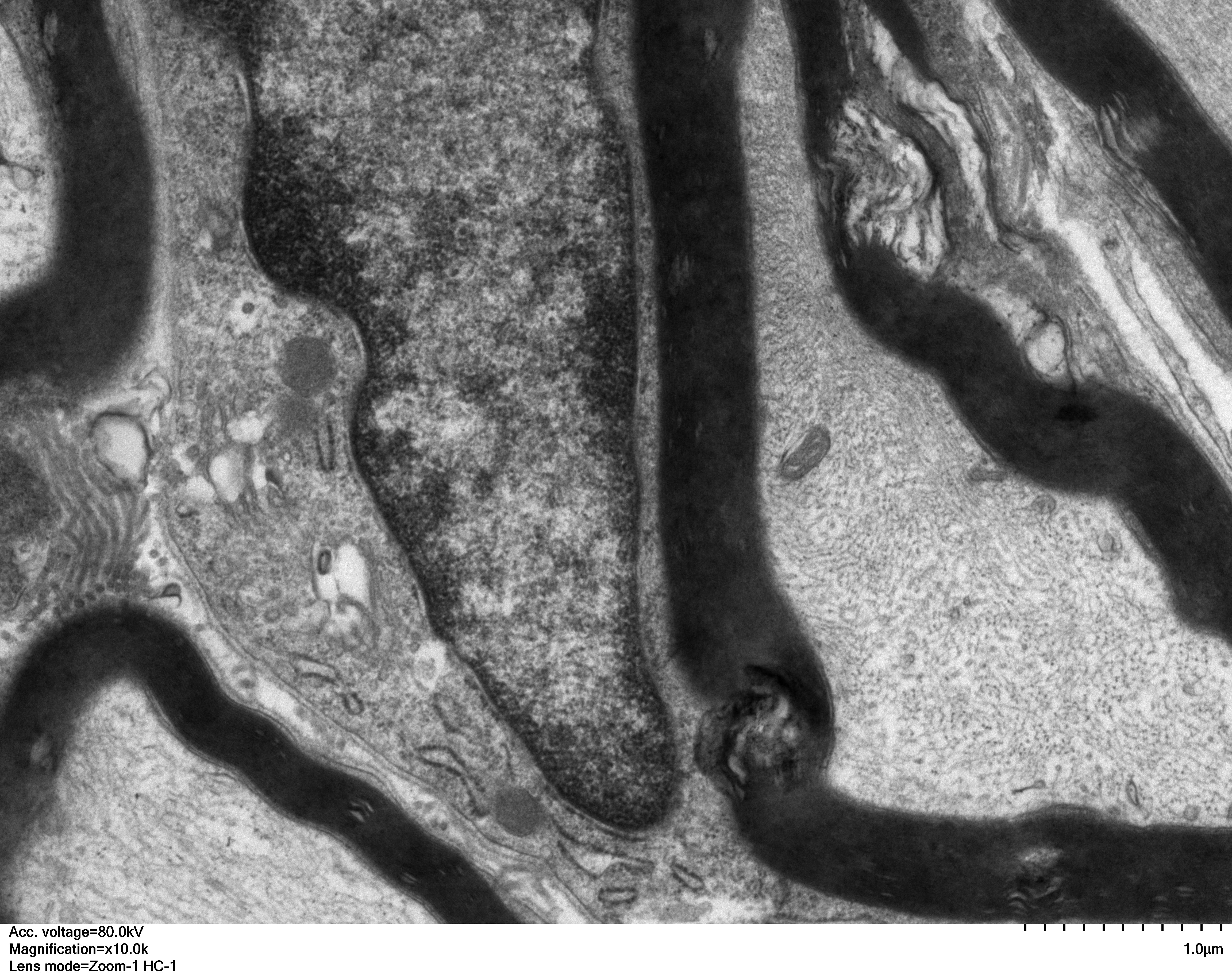

Supplement: Supplementary file 1 [file Data_Sheet_1.zip › Picture/TEM-trigeminal nerve/NA-.jpg]
